# Arginine deprivation alters microglial polarity and synergizes with radiation to eradicate non-arginine-auxotrophic glioblastoma tumors

**DOI:** 10.1172/JCI142137

**Published:** 2022-03-15

**Authors:** Nabil Hajji, Juan Garcia-Revilla, Manuel Sarmiento Soto, Richard Perryman, Jake Symington, Chad C. Quarles, Deborah R. Healey, Yijie Guo, Manuel Luis Orta-Vázquez, Santiago Mateos-Cordero, Khalid Shah, John Bomalaski, Giulio Anichini, Andreas G. Tzakos, Timothy Crook, Kevin O’Neill, Adrienne C. Scheck, Jose Luis Venero, Nelofer Syed

**Affiliations:** 1 John Fulcher Molecular Neuro-oncology Laboratory, Department Brain Sciences, Imperial College, London, United Kingdom.; 2 Department of Biochemistry and Molecular Biology, Faculty of Pharmacy, University of Seville, Seville, Spain.; 3 Instituto de Biomedicina de Sevilla (IBiS), Hospital Universitario Virgen del Rocío (HUVR)/CSIC, University of Seville, Seville, Spain.; 4 Division of Neuroimaging Research, Barrow Neurological Institute, Phoenix, Arizona, USA.; 5 Department of Cell Biology, Faculty of Biology, University of Seville, Seville, Spain.; 6 Center for Stem Cell Therapeutics and Imaging (CSTI), Brigham and Women’s Hospital, Harvard Medical School, Boston, Massachusetts, USA.; 7 Polaris Pharmaceuticals Inc., San Diego, California, USA.; 8 Department of Chemistry, University of Ioannina, Ioannina, Greece.; 9 Institute of Materials Science and Computing, University Research Center of Ioannina, Ioannina, Greece.; 10 Department of Child Health, University of Arizona College of Medicine, Phoenix, Arizona, USA.

**Keywords:** Oncology, Therapeutics, Amino acid metabolism, Brain cancer, Nitric oxide

## Abstract

New approaches for the management of glioblastoma (GBM) are an urgent and unmet clinical need. Here, we illustrate that the efficacy of radiotherapy for GBM is strikingly potentiated by concomitant therapy with the arginine-depleting agent ADI-PEG20 in a non-arginine-auxotrophic cellular background (argininosuccinate synthetase 1 positive). Moreover, this combination led to durable and complete radiological and pathological response, with extended disease-free survival in an orthotopic immune-competent model of GBM, with no significant toxicity. ADI-PEG20 not only enhanced the cellular sensitivity of argininosuccinate synthetase 1–positive GBM to ionizing radiation by elevated production of nitric oxide (˙NO) and hence generation of cytotoxic peroxynitrites, but also promoted glioma-associated macrophage/microglial infiltration into tumors and turned their classical antiinflammatory (protumor) phenotype into a proinflammatory (antitumor) phenotype. Our results provide an effective, well-tolerated, and simple strategy to improve GBM treatment that merits consideration for early evaluation in clinical trials.

## Graphical abstract

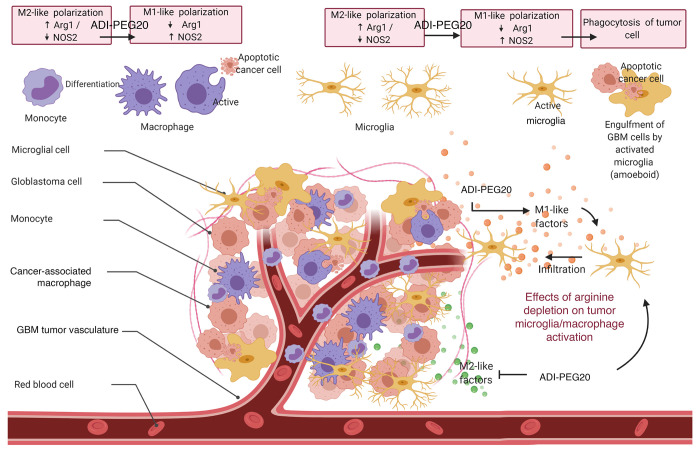

## Introduction

Glioblastoma (GBM) is a highly malignant primary brain tumor for which there have been no improvements in treatment options for almost 2 decades (1–4). Despite the promise of targeted, and more recently, immunotherapeutic approaches, the standard of care for GBM remains unchanged, namely, surgical resection followed by radiotherapy and concomitant temozolomide (TMZ). Treatment resistance and tumor recurrence have been attributed to the invasive nature of GBM growth, which limits the completeness of surgical resection, and to major changes in the tumor microenvironment (TME) (5). Brain-resident microglia and bone marrow–derived macrophages infiltrate the tumor and together form a large part of the brain TME, contributing up to 30% of the tumor mass (6, 7). Activation of microglia/macrophages is a dynamic and tightly regulated process transitioning between a proinflammatory phenotype, characterized by inflammatory antitumor responses, and an antiinflammatory cytoprotective and immunosuppressive phenotype (8). Although the pro- and antiinflammatory microglial states exhibit a spectrum of features, the expression of inducible nitric oxide synthase (iNOS) and arginase 1 (Arg1) has been proposed as a differentiating parameter, where iNOS is expressed by the former and Arg1 by the latter state (8, 9). iNOS and Arg1 both metabolize L-arginine to produce nitric oxide (**˙**NO) and citrulline (via the citrulline/**˙**NO pathway) and L-ornithine and urea, respectively (10, 11). **˙**NO is a critical regulator of host immune responses and ornithine is a precursor for the synthesis of polyamines and prolines, which are important metabolites for proliferation and tumor progression (12–14). In GBM, microglia/macrophages are predominantly polarized to an antiinflammatory phenotype to promote tumorigenesis and create an immunosuppressive TME (15). Thus, strategies that aim to reprogram microglial/macrophage polarity have the potential to provide more successful therapeutic opportunities (16).

Although arginine is predominantly synthesized systemically via the intestinal renal axis by the action of 2 cytosolic enzymes, argininosuccinate synthetase 1 (ASS1) and argininosuccinate lyase (ASL), it can be synthesized locally in cells that express ASS1 and ASL from citrulline and aspartate and hence is considered to be semiessential (10, 11). Since arginine is required by a variety of cancer cells, especially those that are highly proliferating (17, 18), therapeutic arginine deprivation in ASS1-deficient cancers has been examined as a potential anticancer strategy in a number of tumor types, including our own studies in GBM (19–21). In our earlier studies, we demonstrated that 30% of GBM lack the expression of ASS1 and depleting arginine using pegylated arginine deiminase (ADI-PEG20) resulted in cell death in vitro and tumor regression in an orthotopic xenograft model (19–20). GBM cells and tumors expressing ASS1 were unaffected by ADI-PEG20. More recently, we demonstrated that ADI-PEG20 is well tolerated and has promising clinical potential in combination with chemotherapy in a phase I study of recurrent high-grade gliomas that are arginine auxotrophic and ASS1 deficient (22). In cancer, arginine predominantly has an oncogenic role and its immunomodulatory role is hindered. The immunomodulatory role of arginine is mediated not only through its metabolic intermediate, **˙**NO, but also by its direct metabolism by T lymphocytes to promote their antitumor activity (10, 23–25). Arg1-expressing myeloid cells in the TME impair T cell responses by modulating the bioavailability of arginine and inducing immunosuppression (26). Downregulation of Arg1 by pharmacological depletion of extracellular arginine has the potential to restore antitumor responses by local generation of intracellular arginine through the citrulline/**˙**NO pathway via upregulation of ASS1 in T lymphocytes (26).

In the present study, we demonstrate that arginine deprivation using ADI-PEG20 favorably alters the immune microenvironment of ASS1-positive GBM tumors and in combination with ionizing radiation (IR) leads to complete tumor elimination with a highly significant increase in survival. Moreover, we show that tumor destruction is via local generation of cytotoxic peroxynitrites resulting from increased **˙**NO production (via upregulation of iNOS and recycling of citrulline generated by the breakdown of arginine from ADI-PEG20) and the localized production of superoxide anion radicals (O_2_^•−^) in the IR-targeted area (27) in addition to the enhanced phagocytosis by the macrophages/microglia.

## Results

### ADI-PEG20 sensitizes ASS1-positive GBM cells to IR in 3D but not in 2D culture conditions.

**Figure 1:**
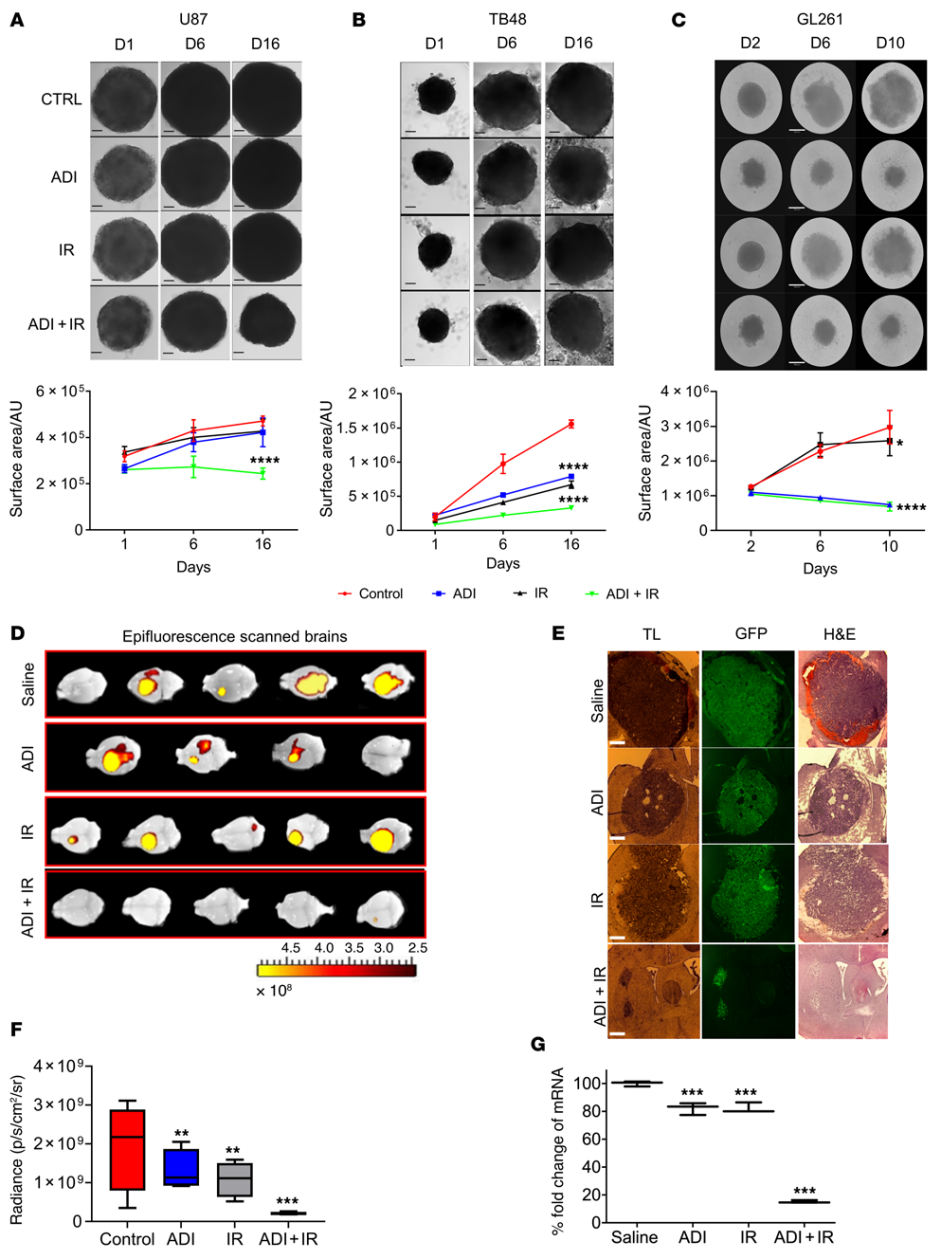
ADI-PEG20 in combination with radiation significantly reduces the growth of ASS1-positive GBM neurospheres and inhibits tumor growth in syngeneic mice. Five thousand cells were plated in low-attachment 96-well plates and incubated for 3 to 8 days to allow formation of neurospheres. Neurospheres were pretreated with ADI-PEG20 (1 μg/mL for human lines and 0.25 μg/mL for mouse line, GL261) for 24 hours before exposure to 2 Gy of ionizing radiation (IR). (**A**–**C**) Images were taken on indicated days after IR treatment and changes in neurosphere surface area measured using ImageJ software (upper and lower panels). (**D**) Epifluorescence (GFP intensity) was measured in whole brains using in vivo image analysis (IVIS). (**E**) Microscopic analysis of representative brain sections: transmitted light (TL), GFP, and H&E staining. Scale bars: 360 μm (**A**–**C**) and 430 μm (**E**). (**F**) Tumor size is represented as total radiant efficiency. (**G**) qPCR expression levels of GFP in tumor sections. The neurosphere results are presented as mean ± SD, *n* = 12. The in vivo results were obtained from 5 animals per group except for animals treated with ADI-PEG20 monotherapy, which only had 4 animals due to the premature death of 1 mouse. Data were analyzed using 1-way ANOVA (**A**–**C** and **F**) or 2-way ANOVA with Tukey’s multiple comparison test with adjusted *P* values reported (**G**). *P < 0.05, **P < 0.01, ***P < 0.001, ****P < 0.0001. AU, arbitrary units.

We previously demonstrated that a subset of GBM is susceptible to arginine deprivation by ADI-PEG20 treatment due to methylation-dependent downregulation of *ASS1* in vitro and in vivo using an orthotopic xenograft model of GBM (19, 20). Interestingly, these studies revealed that ADI-PEG20 potentiated the effects of TMZ in ASS1-positive and hence non-arginine-auxotrophic GBM (20). To explore the effects of arginine deprivation on the radiosensitivity of ASS1-positive GBM in vitro, we combined ADI-PEG20 with radiation in 2D (human lines, U87 and TB48) and 3D (U87, TB48, and mouse cell line, GL261) cultures. ADI-PEG20 had minimal effects on the radiosensitivity of cells in 2D culture (Supplemental Figure 1; supplemental material available online with this article; https://doi.org/10.1172/JCI142137DS1), whereas in 3D culture the combination with radiation significantly reduced neurosphere growth of both human and mouse GBM cell lines (Figure 1, A–C). GL261 neurospheres were also significantly reduced in size with ADI-PEG20 monotherapy (Figure 1C).

### Arginine deprivation sensitizes ASS1-positive GBM to radiation in vivo.

**Figure 2:**
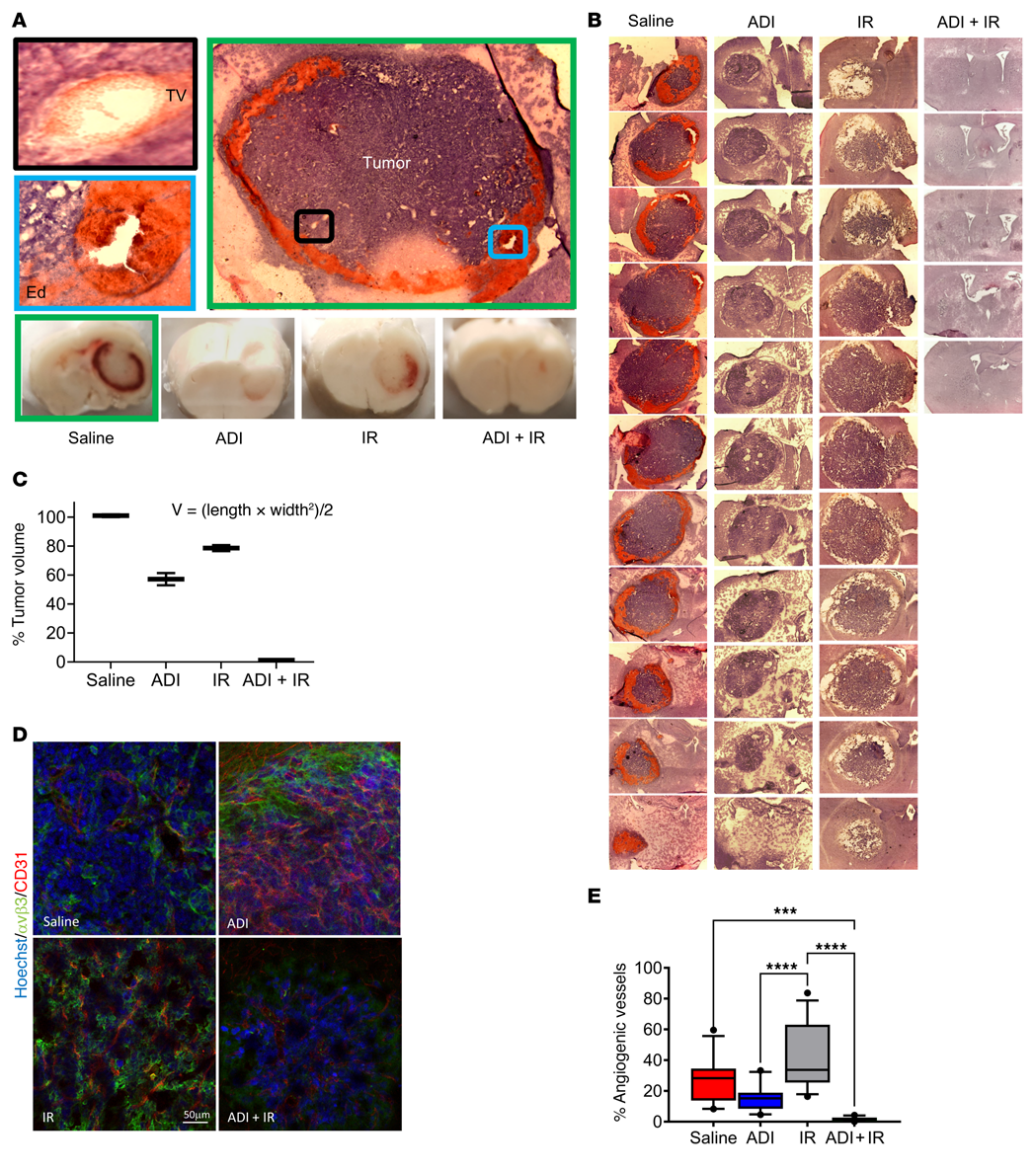
ADI-PEG20 induces significant reduction in ASS1-positive tumor edema and angiogenic vessels and in combination with ionizing radiation drastically reduces GL261-GFP tumor growth. (**A**) H&E-stained fresh-frozen brain sections from saline-treated animals (green box) and cryoblocks from all treatment groups. Vasogenic edema (blue box) (Ed) and intratumor vasculature (black box) (TV) in saline control animals (green box). (**B**) Representative brains from each treatment group were cryosectioned and stained with H&E. (**C**) The percentage tumor volume was measured using the formula *V* = (*L* × *W*^2^)/2, where *L* represents the largest tumor diameter and *W* represents the perpendicular tumor diameter. (**D**) Immunohistochemical analysis of free-floating sections for tumor vasculature using anti-CD31 and –α_v_β_3_ integrin antibodies. Scale bar: 50 μm. (**E**) Percentage of angiogenic vessels in tumor sections as assessed by colocalization of CD31 and α_v_β_3_ staining. Results are representative of 3 animals per group. Note in the combined treatment group only 1 animal had evidence of tumor. ***P < 0.001; ****P < 0.0001. Data were analyzed using 1-way ANOVA.

To further explore the effects observed in 3D culture, we performed in vivo experiments using an immune-competent orthotopic model of GBM utilizing ASS1-positive GL261 cells engineered to express green fluorescent protein (GFP). One week after intracranial injection of cells, mice were randomly sorted into 4 groups of 5 animals and treated with saline (control group), ADI-PEG20 monotherapy, IR monotherapy, or ADI-PEG20 plus IR, as described in the Methods. The experiment was terminated 2 weeks after treatment and animals were analyzed for tumor growth. Single treatment with ADI-PEG20 or IR significantly reduced tumor growth compared with saline-treated, control animals. However, when treatments were combined, complete inhibition of tumor growth was observed in 4 out of 5 animals as evidenced by epifluorescence analysis of the whole brain, GFP fluorescence analysis of brain sections, assessment of GFP mRNA in the tumor, and histopathological analysis of brain sections (Figure 1, D–G). No significant weight loss was observed in any of the groups (Supplemental Figure 2A). Histopathological assessment revealed the typical GBM-associated features of large blood vessels and vasogenic edema in the saline-treated animals that were reduced in all other treatment groups, but the maximal response was observed in the group treated with ADI-PEG20 and IR (Figure 2, A–C). Upon further assessment of tumor vascularization and neovascularization (using anti-CD31 and –α_v_β_3_ integrin antibodies, respectively) we observed clear reduction in both features by ADI-PEG20 compared with saline control animals and maximal reduction with combined therapy (Figure 2, E and E). Together, these results imply that the combination of ADI-PEG20 and IR has significant beneficial effects in ASS1-positive GBM.

### ADI-PEG20 combined with IR eradicates intracranial GBM tumors and enhances survival.

**Figure 3:**
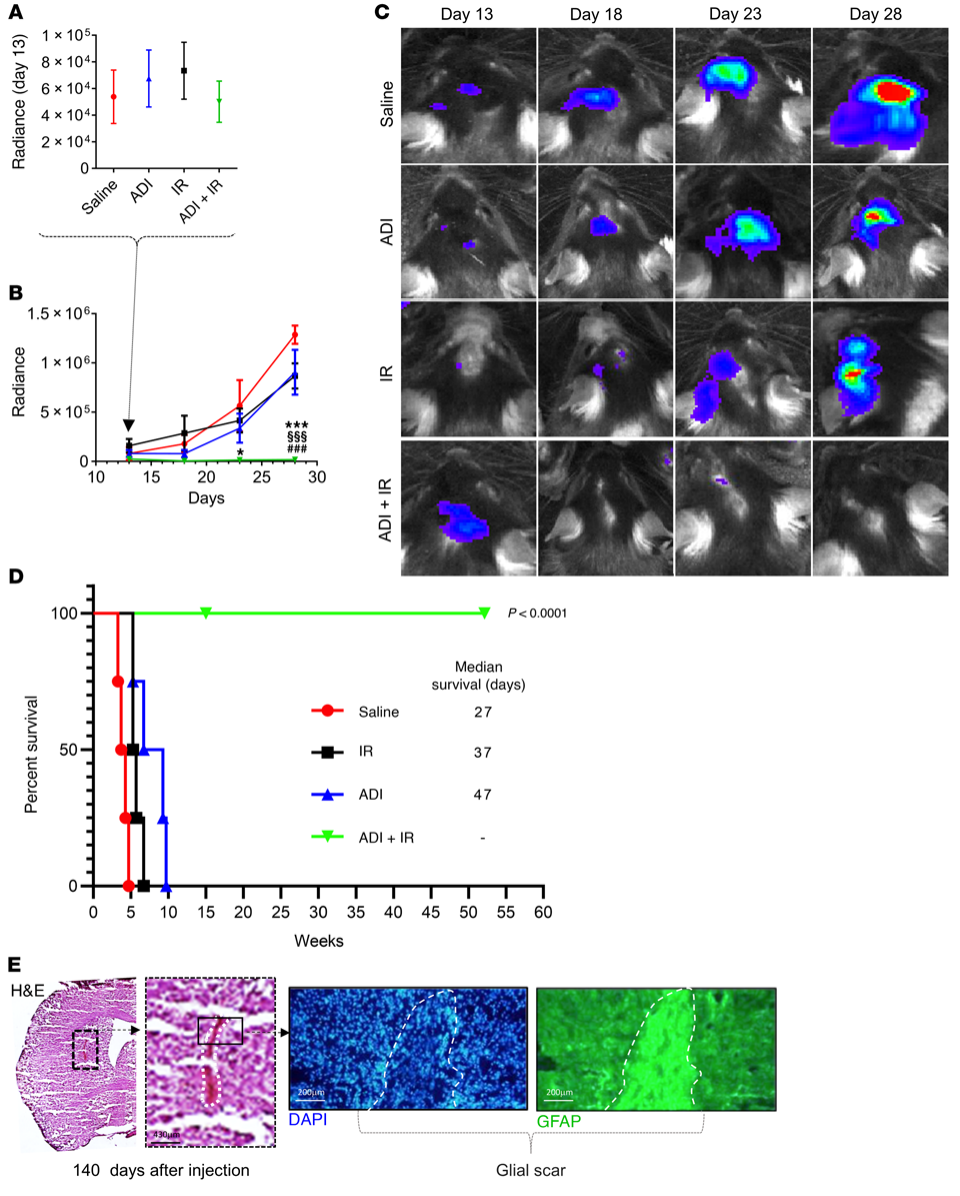
Eradication of GBM intracranial tumors, glial scar formation, and enhanced survival of mice induced by ADI-PEG20 in combination with ionizing radiation. (**A**–**C**) Bioluminescence imaging (BLI) of intracranial tumors in mice using an IVIS Lumina II and Living Image software starting from day 13 after injection of GL261-Luc2 tumor cells. (**D**) Kaplan-Meier survival graph. Median survival times were 27, 47, and 37 days for saline, ADI-PEG20, and ionizing radiation (IR) monotherapy, respectively. Animals treated with combined treatment remained healthy and tumor free beyond 1 year. These animals received IR for 8 weeks and ADI-PEG20 for 13 weeks, after which time treatments were stopped. (**E**) Two animals from this group were culled on day 140 and brain sections were stained with H&E and for GFAP, showing evidence of histologically apparent glial scarring at the tumor site. Scale bars: 430 μm (left); 200 μm (middle, right). Data were analyzed using 2-way ANOVA with Tukey’s multiple comparison test, and adjusted *P* values are reported. ***P < 0.001 (saline vs. ADI-PEG20) + IR; ^§§§^*P* < 0.001 (ADI-PEG20 vs. ADI-PEG20 + IR); ^###^*P* < 0.001 (IR vs. ADI-PEG20 + IR).

Having observed that combination treatment with ADI-PEG20 and IR leads to striking short-term regression of GBM, we extended these studies to examine survival. We used GL261 cells that had been engineered to express luciferase (28) to monitor tumor development and regression in real time. GL261-Luc2 cells were injected intracranially as described previously and tumor growth was evaluated by quantitative bioluminescence imaging (BLI) every 4 to 5 days. Bioluminescence was first detected on day 13 after injection of cells and at this point animals were randomly stratified into 4 groups of 8 animals and treated as described above (saline, ADI-PEG20 monotherapy, IR monotherapy, and combination therapy) (Figure 3A). A steady increase in bioluminescence was observed in saline-treated control animals (Figure 3, B and C). This increase was delayed with ADI-PEG20 and IR alone and tumor growth in these animals generally remained low relative to control animals. Strikingly, animals in the combination group showed no evidence of tumor growth (Figure 3, B and C). Median survival times for animals treated with saline, IR, or ADI-PEG20 were 27, 37, and 47 days, respectively. Animals receiving combination therapy continued to remain healthy and tumor free. No animals experienced any significant weight loss and the group receiving combination treatment gained weight (Supplemental Figure 2B). Animals receiving IR were given a weekly dose of 2 Gy radiation (2 Gy in the tumor-injected left side and 2 Gy in the contralateral side) for a total of 3 weeks. Animals received a total dose of 6 Gy per hemisphere at the end of the experiment. ADI-PEG20 was continued for 13 weeks on a weekly basis and then stopped. With respect to the group that received combination therapy, on day 140, 2 animals were culled, and brains harvested for further analysis while the remaining 2 were monitored with no further treatments. In the culled animals, glial scar tissue was observed at the tumor site, as evidenced by the expression of glial fibrillary acid protein (GFAP) (Figure 3E), consistent with treatment response (Figure 3, B and C). The remaining mice showed no evidence of tumors beyond 50 weeks and were culled thereafter (Figure 3D).

### Arginine deprivation increases microglial/macrophage tumor recruitment and enhances their antitumoral phenotype in vivo.

**Figure 4:**
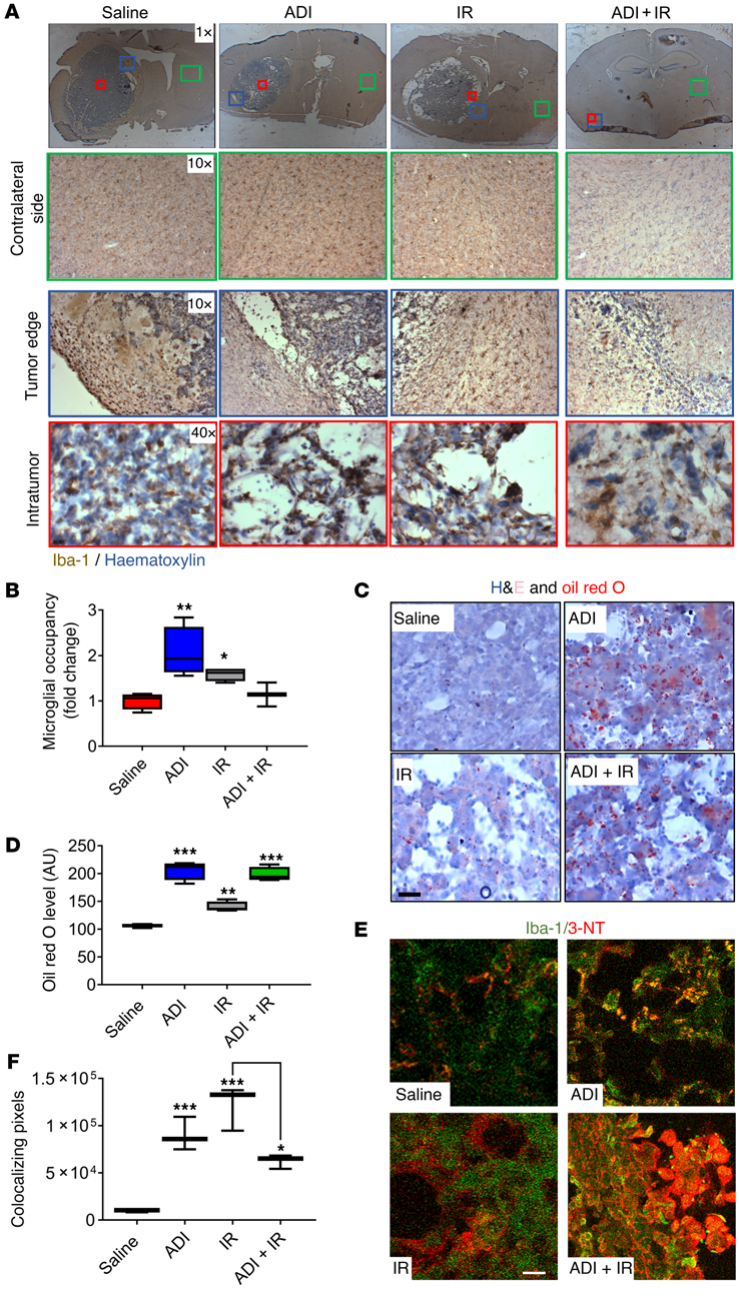
Arginine deprivation increases recruitment of microglia into tumors and enhances their activity and phagocytic phenotype. (**A**) Immunohistochemical evaluation of microglial/macrophage infiltration using Iba-1 staining on the contralateral nontumor side (green box), tumor edge (blue box), and intratumor (red box). (**B**) Intratumoral microglia occupancy. (**C** and **D**) Characterization of microglial phagocytic capacity using H&E and Oil red O staining of tumor sections and quantification of lipid bodies. (**E** and **F**) Assessment of the level of **˙**NO production by microglia by 3-nitrotyrosine (3-NT) and Iba-1 staining of tumor sections. Iba-1 (green), 3-NT (red), colocalization of Iba-1 and 3-NT (yellow). Results were obtained from 5 animals per group. Note in the combined treatment group, analysis was carried out on the single animal showing evidence of tumor. Scale bars: 50 μm. Data were analyzed using 1-way ANOVA. *P < 0.05, **P < 0.01, ***P < 0.001.

To unravel the mechanistic basis for the observed effects with combined therapy, we investigated the GBM TME, an essential component of brain tumors thought to contribute to their maintenance and resistance to therapy. Since microglia/macrophages are critical regulators of the brain microenvironment, we characterized the effect of ADI-PEG20 on this population of cells by immunohistochemistry (IHC) using the pan-microglial marker Iba-1. Glioma-associated macrophages/microglia (GAMM) in brain sections from saline-treated animals exhibited a typical resting-ramified morphology surrounding the tumor mass, with limited infiltration (Figure 4, A and B). In contrast, monotherapy with either ADI-PEG20 or IR significantly increased GAMM infiltration into the tumor mass (Figure 4, A and B). Analysis of GAMM in animals treated with combination therapy was difficult because (a) the absence of tumor in 3 out of 4 treated animals, and (b) the extremely small volume of tumor in the solitary animal with evidence of tumor growth. However, critically, morphology of infiltrating GAMM in the solitary evaluable animal was suggestive of a phagocytic phenotype similar to that with ADI-PEG20 treatment alone (Figure 4A). To explore this further, we stained tumor sections with Oil red O to detect intracellular lipid-like body accumulation, an indicator of phagocytotic capacity. Intracellular lipid-like body accumulation was increased by ADI-PEG20 alone and when combined with IR in the intratumoral area dominated by GAMM infiltration. These results suggest that arginine deprivation switched GAMM activation from a tumor-supporting phenotype to a more phagocytosis-competent and hence tumor-inhibiting phenotype (Figure 4, C and D, and Supplemental Figure 3). **˙**NO produced by activated proinflammatory microglia/macrophages through upregulation of iNOS is associated with cytotoxicity, apoptosis, and bystander antitumor effects (6). Most of the cytotoxicity attributed to **˙**NO is from peroxynitrite, a powerful oxidant formed from the diffusible reaction between **˙**NO and superoxide anion radicals, both of which are simultaneously produced by activated GAMM under proinflammatory conditions (29, 30). Peroxynitrite causes nitrosylation of tyrosine residues (3-NT) in proteins and can be detected by IHC. To seek further verification that arginine deprivation was driving the switching of GAMM function to an antitumor-like phenotype, tumor sections were stained for the presence of 3-NT, an indirect measure of **˙**NO levels and hence of iNOS activity (31). Increased intratumoral 3-NT staining was detected in ADI-PEG20–treated animals and colocalized with Iba-1–expressing cells (Figure 4, E and F). Analysis of sections from combination therapy was again complicated by the small amount of tumor present; however, levels of 3-NT and GAMM colocalization remained significant.

Our data imply that arginine deprivation in non-auxotrophic GBM favorably alters the properties of infiltrating GAMM from a tumor-supporting to a tumor-suppressing role.

### ADI-PEG20 combined with IR has no effect on microglial infiltration in normal brain.

**Figure 5:**
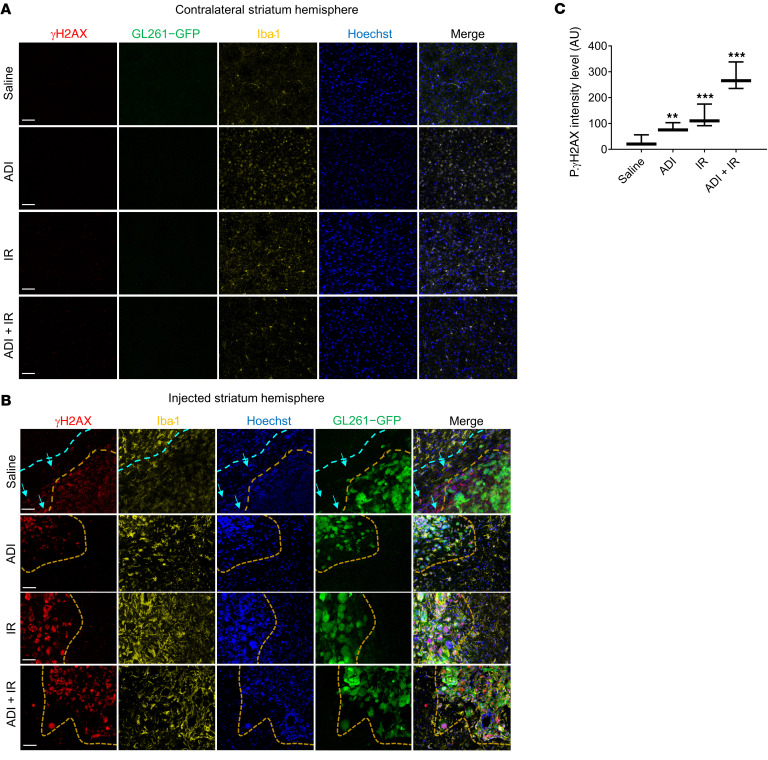
ADI-PEG20 combined with ionizing radiation in vivo significantly increases γ-H2AX in GBM tumors. (**A**–**C**) Immunohistochemical assessment of microglial recruitment and γ-H2AX intensity levels in the contralateral (nontumor) and tumor side of the brains using free-floating tissue sections and quantification of results. γ-H2AX intensity levels were quantified using a Zeiss confocal microscope (Observer Z1) and ZEN 2 (blue edition) software. Iba-1 = yellow, tumor (GFP) = green. Results are representative of 3 animals per group, except for the tumor in the combined treatment animal since only 1 animal had evaluable tumor. Scale bars: 200 μm. Data were analyzed using 1-way ANOVA. **P < 0.01, ***P < 0.001.

To investigate the possible detrimental effects of arginine deprivation and IR on normal brain, we examined the contralateral side of the mouse brain for microglial/macrophage infiltration and for levels of H2AX phosphorylation (γH2AX), a direct measure of IR-induced DNA damage. No evidence of microglial/macrophage infiltration or γH2AX was detected in the contralateral side of the brain in all experimental groups (Figure 5A). However, the level of γH2AX was significantly increased and high levels of GAMM infiltration were observed in the intratumor area after IR or ADI-PEG20. Levels of γH2AX were further elevated when treatments were combined (Figure 5, B and C). Interestingly, γH2AX was detected in areas surrounding the tumor mass (Figure 5B).

### Arginine deprivation activates the antitumor phenotype of GAMM and restores the proinflammatory microenvironment.

**Figure 6:**
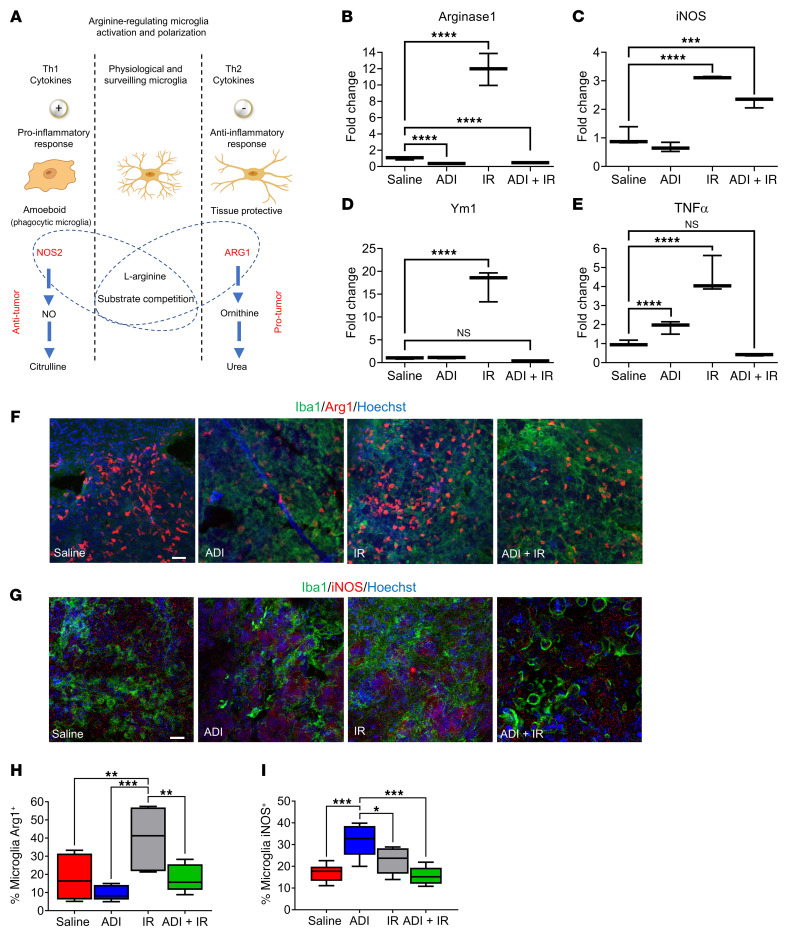
Arginine availability modulates microglial polarization. (**A**) Schematic representation of hypothesized microglial activation and polarization by arginine availability. (**B**–**E**) qPCR analysis of *Arg1*, *iNOS*, *Ym1*, and *Tnfa* expression levels in tumor tissue. (**F**–**I**) Immunohistochemical assessment and quantification of microglial Arg1 and iNOS levels by Iba-1/Arg1 and Iba-1/iNOS costaining of free-floating sections. Scale bars: 100 μm. Data were analyzed using 1-way ANOVA. *P < 0.05, **P < 0.01, ***P < 0.001, ****P < 0.0001. NS, not significant.

L-Arginine has a crucial role in the different microglial/macrophage activation pathways through expression of iNOS and Arg1 enzymes. Since each enzyme negatively regulates the activity of the other (Figure 6A and ref. 32), we next sought to gain insights into the molecular mechanisms that govern the switch in microglial/macrophage polarity upon arginine deprivation in vivo. We carried out qPCR analysis of these key polarization markers together with 2 additional markers, *Ym1* (antiinflammatory) and *Tnfa* (proinflammatory) in RNA from brain sections. Arginine depletion alone significantly downregulated *Arg1* and upregulated *Tnfa* but had no effect on *iNOS* expression (Figure 6, B, C, and E). However, when combined with IR, significant downregulation of *Arg1* and upregulation of *iNOS* was observed but *Tnfa* was downregulated (Figure 6, B, C, and E). Moreover, although *Ym1* expression was not significantly altered in either condition, its downregulation was trending toward significance with combined treatment (Figure 6D). IR significantly upregulated all 4 genes. Of note, qPCR analysis was performed on whole-brain sections and not on isolated tumor samples due to the tumor being barely visible in the combination group. This may explain the results obtained in this group particularly with regard to *Ym1* and *Tnfa* (Figure 6, D and E). To explore whether gene expression changes were specifically attributed to the GAMM population, we costained free-floating tumor sections for Arg1/Iba-1 or iNOS/Iba-1. Although we observed downregulation of *Arg1* and significant upregulation of *iNOS* specifically in the microglia/macrophage population with ADI-PEG20 alone, no differences were observed with combined treatment when compared to the saline control. As stated previously, we believe this may be reflective of the small tumor size in the combination treatment group. Our results demonstrate that arginine deprivation specifically altered the expression of *Arg1* and *iNOS* in the GAMM population, thereby altering their polarity toward a phenotype more capable of targeting the GBM tumor (Figure 6, F–I).

To further validate these results in vitro, we treated microglial cells (BV2) with ADI-PEG20 or cultured them in conditioned medium from GBM cells (GL261) that had been treated with ADI-PEG20 and analyzed them for the expression of *Arg1* and *iNOS* by qPCR. *Arg1* expression was downregulated under conditions where arginine was degraded by ADI-PEG20 (Supplemental Figure 4A). When GBM neurospheres (TB48 and GL261) were cocultured with microglial cells and exposed to ADI-PEG20, microglial infiltration was increased and a corresponding reduction in neurosphere growth was observed (Supplemental Figure 4B, upper and lower panels). We then performed combination experiments in vitro where we challenged microglial cells with 100 ng/mL ADI-PEG20 and 2 Gy of IR. Radiation alone induced a 5-fold increase in *Arg1* expression, which was restored to basal levels when combined with ADI-PEG20 (Supplemental Figure 4C). The upregulation of additional recognized markers of proinflammatory macrophages/microglia (CD86, CD40, and IL-12) was also observed in response to both ADI-PEG20 alone and in combination with IR (Supplemental Figure 5).

**Figure 7:**
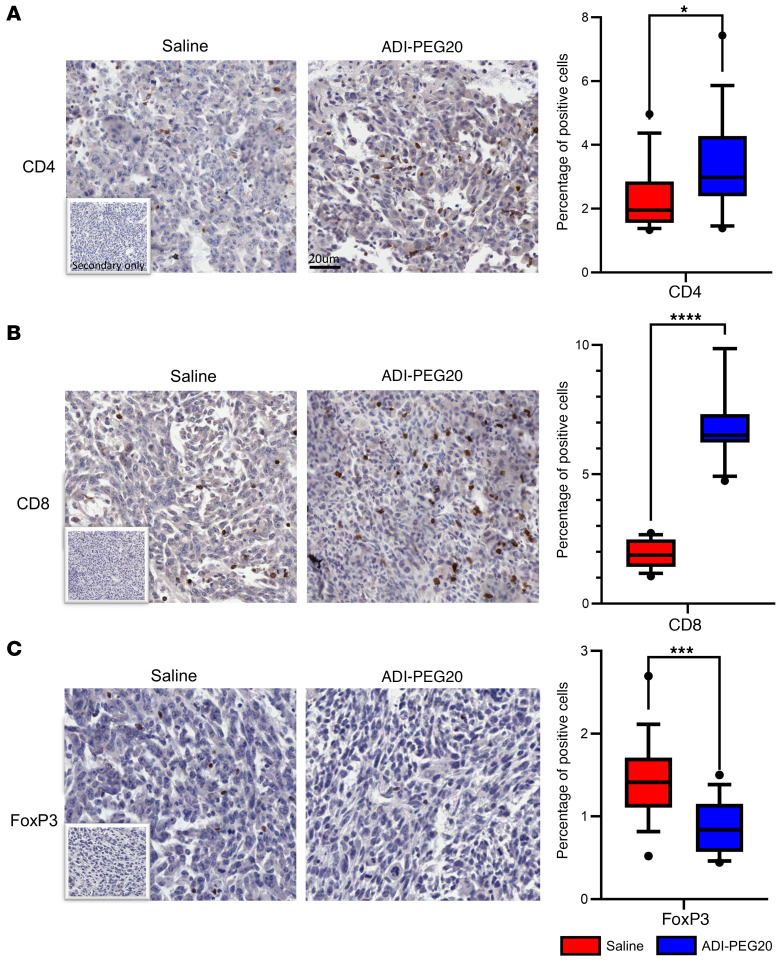
Arginine deprivation reverts the immune-suppressive microenvironment. (**A**–**C**) Immunohistochemical assessment and quantification of CD4^+^, CD8^+^, and FOXP3^+^ T cells. Tissue sections were stained using an Aperio AT2 slide scanner and analyzed using QuPath (v1.2.2) based on 5 randomly selected regions. Scale bar: 20 μm. Data were analyzed using a nonparametric Mann-Whitney *U* test. *P < 0.05, ***P < 0.001, ****P < 0.0001.

Since the expression of *Arg 1* in the TME contributes to immunosuppression by impairing T cell responses through modulating the bioavailability of arginine (26), we investigated whether pharmacological degradation of arginine was able to revert this feature of immunosuppression. To address this, we performed IHC on tumors from saline- and ADI-PEG20–treated animals to look for the presence of CD4^+^, CD8^+^, and FoxP3^+^ regulatory cells. We observed a significant increase in the number of CD4^+^ and CD8^+^ T cells and a corresponding decrease in FoxP3^+^ regulatory cells with ADI-PEG20 treatment (Figure 7, A–C).

### Microglial/macrophage protumor factors are highly expressed in GBM.

**Figure 8:**
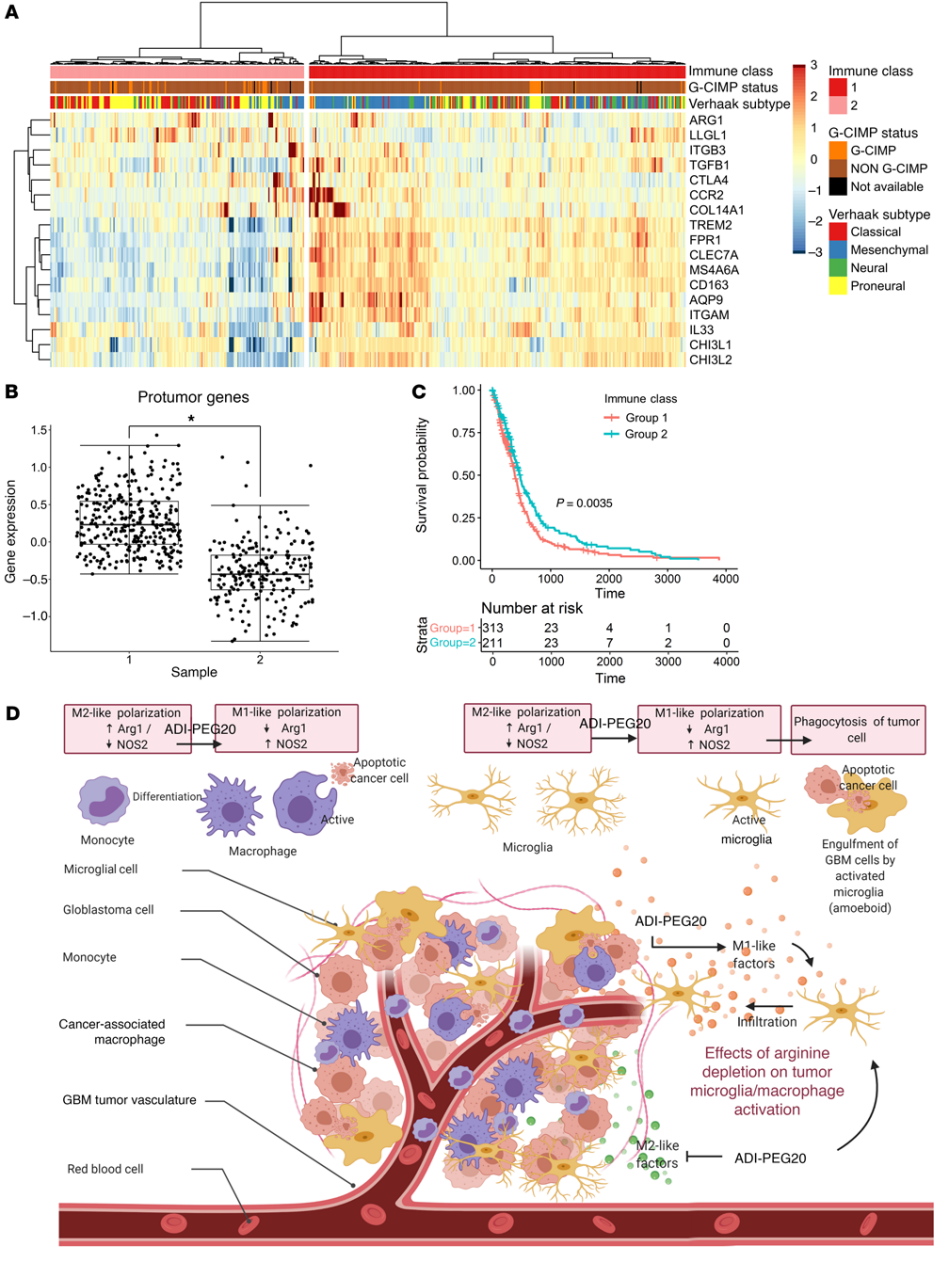
Hierarchical clustering shows that the expression of protumor microglial genes is associated with GBM subtypes. (**A**) Heatmap and dendrogram showing the expression of protumor microglial genes with tumor molecular phenotypes. (**B**) Box-and-whisker plot showing expression of protumor microglial genes in the 2 clusters. (**C**) Kaplan-Meier plot showing patient survival between the 2 clusters. (**D**) Schematic summary of the effect of arginine depletion on tumor microglial/macrophage activation.

Since the presence of microglia/macrophages in brain tumors is thought to support tumor growth and maintenance, we investigated whether this was reflected in the gene expression profile of clinical cases of GBM. We generated a list of microglial/macrophage protumor genes from published manuscripts and interrogated The Cancer Genome Atlas (TCGA) database for their expression (Supplemental Table 1 and ref. 33). Hierarchical clustering of TCGA gene expression data indicated 2 distinct populations. Cluster 1 had much higher expression of protumor genes (Figure 8, A and B; *P <* 0.05) and was associated with poorer survival (Figure 8C; *P <* 0.05). A χ^2^ test revealed significant overrepresentation of the mesenchymal and neural GBM subtypes in cluster 1 (Figure 8A and Supplemental Table 2; *P <* 0.05). Schematic summary of the effect of arginine depletion on tumor microglial/macrophage activation is depicted in Figure 8D.

### ADI-PEG20 combined with IR eradicates CT-2A orthotopic GBM tumors.

**Figure 9:**
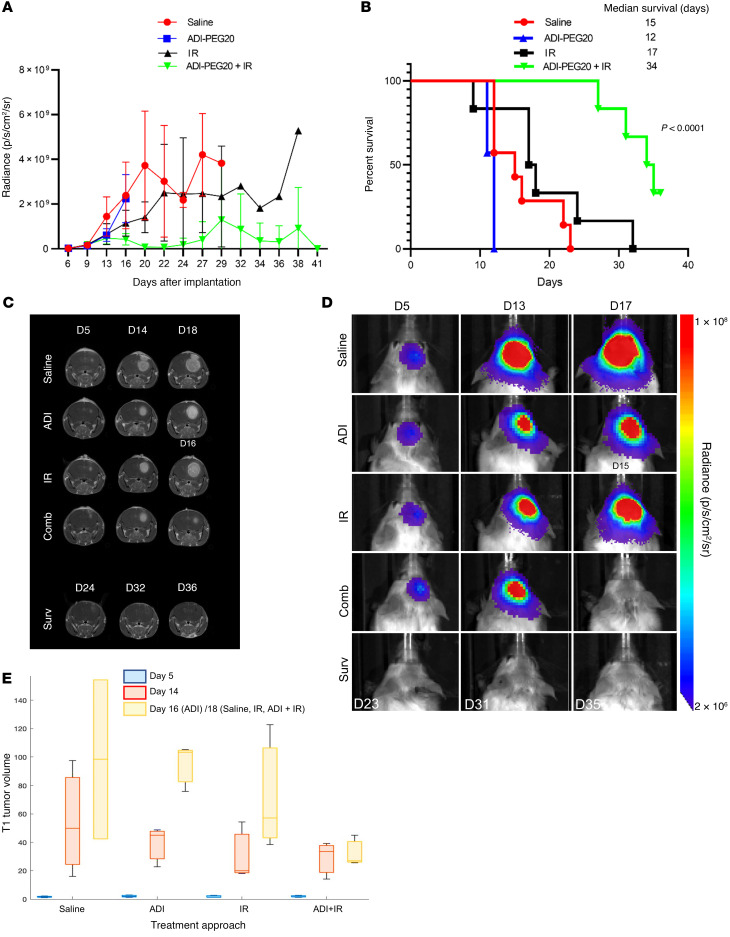
ADI-PEG20 combined with ionizing radiation eradicates CT-2A orthotopic GBM tumors. (**A**) Bioluminescence imaging (BLI) of intracranial tumors in mice using an IVIS Spectrum in vivo imaging system and Living Image software starting from day 5 after injection of CT-2A tumor cells. (**B**) Kaplan-Meier survival graph. Animals administered combined treatment remained healthy and tumor free at time of harvest. Three mice in each treatment group were additionally analyzed by MRI at early (5 days), mid (14 days), and late (16 days for ADI animals and 18 days for other groups) time points after intracranial injection of cells, and additional images were obtained for animals in the combination group. ADI animals were imaged earlier because they showed signs of distress. (**C** and **D**) Representative MR and BL images of mice in each group. (**E**) Box-and-whisker plot of the calculated T1 tumor volumes at early, mid, and late MRI time points.

**Figure 10:**
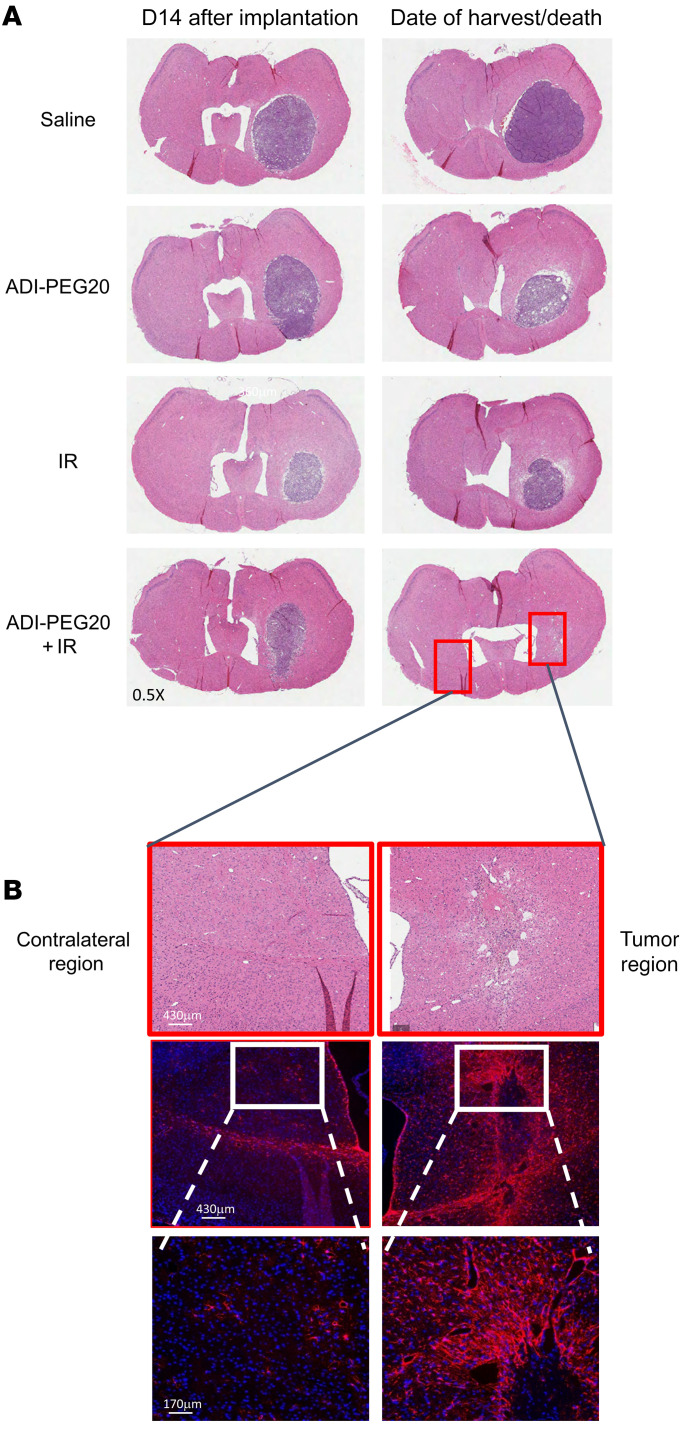
Immunological assessment of CT-2A tumors in mice treated with ADI-PEG20 combined with ionizing radiation. (**A**) Representative H&E images of mouse brains at the mid time point and at time of death/harvest. (**B**) Representative GFAP staining of brains in combined treatment group showing contralateral and tumor region at late harvest time point. Time points: Early, 5 days after implantation and before treatment; Mid, 14 days after implantation and after 1 round of treatment; Late, 16 (ADI animals) and 18 days (all other groups) after implantation and after 2 rounds of treatment. DAPI was used as a nuclear counter stain (blue). Scale bars in **E**: 430 μm (top, middle); 170 μm (bottom).

To further assess the validity and potential clinical translation of our results, we repeated the efficacy studies in the CT-2A syngeneic GBM model. This model accurately recapitulates many features of human GBM histology (34). We first confirmed expression of *ASS1* mRNA in these cells by qPCR before proceeding with the in vivo experiments (Supplemental Figure 6). The in vivo experimental plan is schematically represented in Supplemental Figure 7. Essentially, groups of 10 animals were used for each treatment and tumor establishment and growth were assessed by BLI. Three animals in each group were analyzed by MRI at early, mid, and late time points to detect tumor growth before treatment, after 1 and 2 rounds of treatments, respectively. Three animals from each group were also harvested at the mid time point for H&E analysis. Additional MRI images were taken of animals in the combination group after the third, fourth, and fifth round of treatments. BLI indicated reduced tumor growth in animals treated with ADI-PEG20 in combination with IR and these animals had longer medium survival times (Figure 9, A, B, and D). These results were confirmed by MRI, indicating the presence of tumors in the control and single-treatment groups, with complete tumor eradication seen only in the combination group (Figure 9C). Figure 9E shows a box plot of the calculated T1 tumor volumes at the early, mid, and late MRI time points, indicating reduced tumor growth when ADI-PEG20 is combined with IR. Six out of 7 mice in this group had no visible tumors. A representative H&E image (Figure 10A) shows the presence of tumors in all groups prior to treatment and in the single-treatment groups at time of death. No tumors were observed in the combination group at the time of harvest. Animals in this latter group remained healthy, but exhibited more than 10% weight loss and were humanely killed in line with ethical considerations of the Barrow Neurological Institute, where these experiments were conducted (Supplemental Figure 8). Enhanced gliosis, an indication of scar formation, was observed in the tumor region of these mice, as demonstrated by strong GFAP staining, similar to our observation in the GL261 tumor model (Figure 10B).

## Discussion

Arginine auxotrophy in GBM is common and results from epigenetic transcriptional silencing of *ASS1* (19). This has been shown to be therapeutically exploitable using the arginine-depleting agent ADI-PEG20 in vitro, in vivo, and in a phase I clinical trial (19, 20, 22). However, most studies have reported that ADI-PEG20 monotherapy has no cytotoxic or cytostatic effects against GBM cells that express *ASS1* due to the absence of CpG island methylation. In this study, we demonstrate that arginine depletion when combined with IR is a highly effective therapeutic strategy in non-arginine-auxotrophic (ASS1-positive) GBM and we present evidence that the ability of ADI-PEG20 to potentiate the effects of radiation occurs predominantly via **˙**NO production and its effects on the TME, specifically by driving changes in the GAMM population toward a tumor-suppressing phenotype.

As monotherapy, ADI-PEG20 has been most effective when tumors are deficient in ASS1, the rate-limiting enzyme in the urea cycle that produces L-arginine. ASS1 loss has been associated with an aggressive tumor phenotype, both in preclinical and clinical studies (17, 18). The reason(s) for this are not entirely clear, but appear to be due, at least in part, to rapidly growing cancers preferring to shunt citrulline together with aspartate to form nucleotides, instead of arginine and urea. However, when combined with another therapeutic modality, ASS1 deficiency may not be as important as with ADI-PEG20 monotherapy. For example, the ASS1-positive breast cancer cell line MCF-7 is resistant to ADI-PEG20 monotherapy, but the response to ADI-PEG20 coupled with radiation therapy is synergistic (35). Indeed, ADI-PEG20 plus TMZ is at least additive in a xenograft model, in both ASS1-positive and -negative tumors (20).

In vivo experiments to evaluate the effect of ADI-PEG20 combined with IR in ASS1-positive GBM yielded striking results, where we observed highly significant tumor responses compared with control animals and monotherapy groups, as evidenced by epifluorescence, BLI, measurement of GFP histopathology, and most importantly, survival. The striking inhibition of GBM growth produced by combined ADI-PEG20 and IR was seen after 2 doses (2 consecutive weeks) of combined therapy. Importantly, an additional 6 weeks of combined therapy (once weekly) not only induced complete tumor response but was reflected in remarkable increases in survival. Whereas median survival of the control group was approximately 4 weeks, it was approximately 5 and approximately 7 weeks for animals treated with monotherapy IR or ADI-PEG20, respectively. More than a year after GBM implantation, animals that received combined therapy remained alive and healthy with no evidence of GBM growth and in the absence of continued treatment. Furthermore, animals treated with combination therapy showed histological evidence of a glial scar at the tumor site when sampled at 15 weeks, but not viable tumor cells. This was verified by the expression of GFAP and the appearances are consistent with complete pathological response. These striking results were reproduced with both BLI and MRI in an additional orthotopic model of GBM using CT-2A cells. Our data, therefore, imply a therapeutic synergy between arginine depletion and IR, and suggest that enhanced production of **˙**NO and changes in the TME mediate the striking, potentially curative effects observed in vivo.

Growth of tumors in the saline-treated control group was associated with large-volume vasogenic edema and extensive neovascularization, typical of GBM. Medical management of vasogenic edema relies on corticosteroids such as dexamethasone, with all the accompanying problems resulting from their long-term use. One of the important therapeutic goals of GBM management is to achieve steroid independence, and using histopathology and CD31 and α_v_β_3_ integrin IHC we show that arginine depletion greatly reduces vasogenic edema and neovascularization. This would be consistent with prior studies examining the role of ADI-PEG20 in the vasculature. The antiangiogenic activity of ADI appears due, at least in part, to distortion of actin filaments, thus disabling the ability of blood vessels to bud, blossom, and grow. Note that this disruption occurs in highly ASS1-proficient endothelial cells. ADI-PEG20 also inhibits hypoxia-inducible factor (HIF), primarily HIF-1α, in both ASS1-proficient and -deficient tumors (36). This was associated with decreased vascular endothelial growth factor (VEGF), an inducer of blood vessel growth. These findings were replicated in mouse xenograft models, and were also associated with decreased blood vessel perfusion. HIF has been implicated in the pathogenesis of GBM (37). Furthermore, high HIF-1α levels decrease TMZ responsiveness, thus abrogating a primary postsurgical treatment for GBM (38). As such, ADI-PEG20 may be having an antitumoral effect, at least in part, from its anti-HIF effects. Translation of these initial observations into the clinical setting would identify arginine deprivation as a viable steroid-sparing strategy for GBM.

Arginine deprivation represents a metabolic vulnerability in arginine auxotrophs but has limited cytotoxic or cytostatic effects in arginine non-auxotrophs (24). In arginine non-auxotrophs, citrulline produced from the degradation of arginine by ADI-PEG20 is recycled via the citrulline/**˙**NO pathway and enhances the formation of **˙**NO, a hydrophobic and chemically inert molecule (39, 40). **˙**NO can permeate cell membranes and be transported to cellular compartments where it exerts paracrine functions in the vascular wall (41). However, when it encounters superoxide anion radicals (O_2_^•−^), peroxynitrite anions (ONOO^–^) are generated at a diffusion-controlled rate of approximately 1.0 × 10^10^ M^−1^s^−1^ (42) which is higher than the superoxide dismutase–catalyzed rate of superoxide anion production (43). Peroxynitrite is a highly toxic compound because it is a potent oxidant that can oxidize and lead to lipid and protein nitration. Absorption of IR by living cells can generate reactive oxygen radicals such as superoxide radicals through the radiolysis of cellular water (44). We therefore hypothesized that contrary to the current perception that ADI-PEG20 is only effective in arginine auxotrophs (45), albeit only moderately as a monotherapy, it can also be potent in non-arginine-auxotrophic tumors through generation of **˙**NO by the recycling of citrulline via the citrulline/**˙**NO pathway. In arginine-depleting conditions, NOS has higher affinity than Arg1 for L-arginine, thus favoring the subsequent formation of cytotoxic peroxynitrite species (46). Therefore, downregulation of *Arg1* and upregulation of *iNOS* in arginine-limiting conditions can further enhance the levels of **˙**NO. In that vein, our results clearly demonstrated the production of toxic peroxynitrite as detected through 3-NT staining in both ADI-PEG20– and combination-treated animals.

To further explore the mechanistic basis for our observed results, we examined the tumor-associated microglia/macrophages more closely. In control saline-treated mice, we observed a characteristic morphological feature of resting-ramified microglia at the periphery of the tumor, with marginal tumor infiltration. Although both ADI-PEG20 and IR monotherapy increased tumor microglial/macrophage infiltration, their expression of Arg1 differed. ADI-PEG20 decreased microglial Arg1 expression and these microglia/macrophages exhibited a phagocytic phenotype, as evidenced by an accumulation of lipid bodies. The opposite was observed with IR, i.e., increased Arg1 expression and reduced lipid-like body accumulation. Combined treatment also reduced Arg1 expression, upregulated iNOS expression, and increased lipid-like body accumulation compared with control animals. These results suggest that depletion of arginine inhibits the microglial/macrophage protumor response induced by IR and subsequently promotes a phagocytic phenotype. These results are consistent with previous studies that reported that *Arg1* expression in microglia of GL261 murine gliomas occurs later during tumor growth and is independent of microglial infiltration into gliomas (47). Furthermore, in 3D cocultures of GBM and microglia, increased infiltration of microglia was observed in both mice and human GBM cells (GL261 and TB48) only in arginine-depleted conditions. Additionally, *Arg1* expression of microglial cells cultured in fresh media and GBM-conditioned medium was significantly reduced only in the presence of ADI-PEG20, where arginine was degraded. Together, our data demonstrate that arginine depletion reduces the level of *Arg1* in resident microglia and increases their infiltration and phagocytic capacity (Figure 8D, Schematic summary). Moreover, Arg1 downregulation resulted in elevated levels of cytotoxic CD8^+^ T cells and a corresponding reduction in immunosuppressive T regulatory cells (FoxP3^+^) in the TME. These features have been linked to a good prognosis in various cancers types, including GBM (48).

We used a curated list of proinflammatory (M2-like) genes from published literature to tease apart microglial phenotypes using TCGA GBM tumor gene expression data (33). This gene list clearly defined the mesenchymal GBM subtype as having a strong immune signature, which corresponds with their aggressive and least therapeutically responsive characteristics (49).

GBM tumors are highly infiltrated with macrophages/microglia whose plasticity makes them an attractive therapeutic target. Our study provides compelling evidence that arginine deprivation combined with IR is a promising immunotherapeutic strategy for the treatment of ASS1^+^ GBM, which operates by enhancing the production of cytotoxic levels of peroxynitrite and selectively modulating GAMM to become tumor attacking.

## Methods

### Cell lines

The U87 cell line was purchased from ATCC and the patient-derived primary GBM cell line TB48 was generated in house as previously described (19). The GL261 cells expressing GFP (GL261-GFP) and BV2 cells were obtained from Joseph Bertrand (Karolinska Institute, Solna, Sweden). All cells, except TB48, were maintained in DMEM supplemented with 10% fetal bovine serum (FBS). TB48 were maintained in DMEM/F12 supplemented with 10% FBS. Cells were regularly tested for mycoplasma contamination using the Venor GeM mycoplasma detection kit (Minerva Biolabs GmbH). DMEM, DMEM/F12, and FBS were purchased from Thermo Fisher Scientific. ADI-PEG20 was obtained from Polaris Pharmaceuticals Inc.

### Colony formation assay

Cells were seeded into 6-well dishes at 500 cells/well and incubated overnight in complete medium. The next day, the medium was changed and cells were treated with 1 μg/mL ADI-PEG20 and irradiated with 2, 4, or 8 Gy, with no further media changes. At various exposure times, cells were washed with phosphate-buffered saline (PBS) and fixed in 100% methanol for 10 minutes at –20°C. Colonies were visualized by staining with a solution of 5% Giemsa, 25% methanol, and 70% PBS and counted as previously described (50).

### 3D neurosphere formation

Five thousand cells/well were seeded in low-attachment 96-well plates (Thermo Fisher Scientific) and cultured for 3 to 8 days to allow the neurospheres to develop. The neurospheres were then treated with ADI-PEG20 (1 μg/mL for human cells and 0.25 μg/mL for mouse cell lines) for 24 hours before being irradiated with 2–4 Gy and allowed to grow for a further 10–15 days. Images were captured and sphere size measurements analyzed using ImageJ software (NIH) as per the manufacturer’s instructions.

### Establishment of orthotopic GBM tumors

#### GL261.

Five- to 7-week-old wild-type littermate mice on the C57BL/6J background were purchased from The Jackson Laboratory. Animals were housed under a 12-hour light/12-hour dark cycle with free access to food and water. Mice were anesthetized with a mixture of ketamine (50 mg/kg, Ketamidor, Richter Pharma) and medetomidine (10 mg/kg, Domtor, Ecuphar) i.p. and preemptively given analgesia s.c. with buprenorphine (0.05 mg/kg, Bupaq, Richter Pharma). Once surgical anesthesia was confirmed, mice were placed into a stereotactic frame and secured with mouse-adapted teeth and ear bars. Ophthalmic gel drops were applied to their mice eyes to prevent drying-derived eye disorders. A midline incision was made to expose the skull. Injection site coordinates (+0.2 mm anterior, –2.3 mm lateral from bregma) were previously determined and an approximately 1-mm-diameter craniotomy was made over the left brain hemisphere using a 0.8-mm diameter drill bit connected to a handheld dental drill. Skull remains were removed carefully to avoid dura mater puncture. Injection of cells (GL261-GFP and GL261-Luc2) was carried out using a 10 μL Hamilton 701N syringe with a borosilicate capillary glass attached to the tip (Harvard Apparatus). Capillary glass was used in order to minimize inflammatory responses derived from injection trauma. Capillary glass was then moved in the ventral coordinate (–3.1 mm) until the desired striatum location was reached. Cells (2 × 10^5^) were slowly injected in a volume of 2 μL. After removal of the Hamilton syringe, the skull cavity was restored with dental cement and the skin was sutured. Postsurgical mice were injected s.c. with 50 μL of atipamezol (0.5 mg/mL) (Nosedorm, Karizoo) and 50 μL of 5% dextrose solution, the former to antagonize the anesthetic effects and the latter to alleviate dehydration and starvation after surgery.

#### CT-2A.

Eight-week-old female B6(Cg)-*Tyr^c-2J^*/J mice (albino variant C57BL/6J) were purchased from The Jackson Laboratory. Animals were housed under a 12-hour light/12-hour dark cycle with free access to food and water. Cells were harvested and resuspended in DMEM containing no FBS at a concentration of approximately 1 × 10^7^ to 2 × 10^7^ cells/mL. Anesthesia was achieved by isoflurane inhalation. Once the animal was fully anesthetized, the incision site was shaved and sterilized with ethanol and povidone-iodine. The animal was then placed on the operation stage, with a stereotactic frame permitting access to the head while maintaining the animal in place. An ophthalmic lubricating ointment was applied to the eyes to prevent drying. An incision along the midline of the head was made with the use of a sterile scalpel. A burr hole was then made in the right hemisphere of the brain approximately 2.3 mm to the right of the sagittal suture and approximately 0.1 mm below the coronal suture. A 10 μL gas-tight microsyringe (Hamilton Neuros 1700 Series) with a 31-gauge needle was inserted to a depth of 3 mm and left in place for 1 minute. After 1 minute, the syringe was withdrawn to a depth of 2.6 mm and 2 μL of tumor cell suspension was injected over a 3-minute period with the use of an UMP3t-1 UltramicroPump 3 (World Precision Instruments). Once the injection sequence was complete, the syringe was left in place for an additional minute before slowing being withdrawn. The burr hole was then closed with bone wax and the incision site was immediately closed with sterile Vicryl sutures. Postsurgery mice were given an i.p. injection of Convenia (8 mg/kg; Zoetis) for antibiosis and a subcutaneous injection of 72-hour time-release buprenorphine SR (0.5–1 mg/kg; Zoopharm LLC) as an analgesic.

### Efficacy studies

#### GL261.

To demonstrate therapeutic efficacy, orthotopic tumors were generated using GL261-GFP cells. Fifteen days after surgery, animals were randomly stratified into 4 groups of 5 mice and treated with saline (control group), ADI-PEG20, IR, or ADI-PEG20 plus IR. The radiation group received 4 × 2 Gy (2 Gy in the tumor-injected left side and 2 Gy in the contralateral side) over 2 weeks and received a total of 8 Gy. The X-ray irradiator operated at 100 kV and a dose rate of 1 Gy/min (Philips MU 15F). ADI-PEG20 (5 IU) was administered intramuscularly once per week for the duration of the study. For the combined group, ADI-PEG20 was administered 16 hours before radiation. The experiment was terminated 2 weeks after treatment, i.e., 28 to 30 days after surgery. Mice were deeply anesthetized prior to transcardial perfusion of 4% paraformaldehyde solution. Brains were removed, fresh frozen, and sequential 30-μm coronal sections were analyzed by IHC and used for RNA extraction.

#### CT-2A.

To demonstrate therapeutic efficacy in another orthotopic model of GBM, CT-2A GBM cells expressing luciferase were used. These studies were performed at the Barrow Neurological Institute. Five days after surgery, bioluminescence measurements were acquired and animals were divided into 4 groups of 10 animals each with equal tumor size distribution (average and variance). Randomization occurred in a blinded fashion. The treatment groups consisted of saline (control group), ADI-PEG20, IR, or ADI-PEG20 plus IR. MRI images were obtained in 3 mice from each group in addition to BLI before treatments were initiated and repeated throughout the study. Mice were treated with intramuscular injections of ADI-PEG20 or saline starting on day 7 after implantation and continuing weekly throughout the study. Sixteen hours after each treatment with saline or ADI-PEG20, mice were anesthetized with isoflurane and positioned in the RS 2000 X-Ray Biological Irradiator (Rad Source Technologies) operating at 160 kV such that the radiation groups received 4 Gy of radiation at 3 Gy/min to only their head and the groups without radiation received none. On day 13, 3 mice from each group were culled. One brain was flash frozen in Tissue-TEK Optimal Cutting Temperature compound (Sakura Finetek) and 2 were fixed via transcardial perfusion with 4% paraformaldehyde to observe tumor progression and microenvironment mid-way through the treatment. The remaining mice were followed with MRI and BLI until they became symptomatic, at which point they were humanely euthanized and either fresh or fixed tissue was collected for analysis.

### Survival studies

For survival studies, orthotopic tumors were generated using GL261-Luc2 cells so that tumor development and regression could be monitored in real time (28). Tumor growth was evaluated by detection of bioluminescence every 4 to 5 days using BLI. Bioluminescence was first detected on day 13 after injection of cells and at this point animals were stratified into 4 groups of 8 animals and treated as described above for the efficacy studies (saline, ADI-PEG20 monotherapy, IR monotherapy, and combination therapy) (Figure 3A). On day 28, 4 animals from each group were sacrificed for pathology and IHC studies while treatments were continued on the remaining animals for survival studies. Animals receiving combination therapy remained healthy and tumor free throughout the study and received a weekly dose of 2 × 2 Gy radiation for a total of 8 weeks, reflecting a total radiation dose of 32 Gy. ADI-PEG20 was continued for 13 weeks on a weekly basis and then stopped. On day 140, 2 animals were culled, and brains harvested for further analysis while the remaining 2 were monitored with no further treatments. Kaplan-Meier analysis was carried out to assess statistical significance.

### BLI

#### GL261 mice.

All in vivo bioluminescence and fluorescence measurements were carried out using the IVIS Lumina II and Living Image Software (Perkin Elmer). For GL261-GFP mice, brains were analyzed after termination of the study. For GL261-Luc2, sequential bioluminescence measurements were taken in live animals following i.p. injection of 100 μL of D-luciferin (Sigma-Aldrich) followed 5 minutes later by an i.p. injection of low-dose ketamine.

#### CT-2A mice.

In vivo BLI was carried out using an IVIS Spectrum in vivo imaging system (Caliper Life Sciences) coupled to the data acquisition Living Image software (Xenogen Corp.). Sequential bioluminescence measurements were taken in live animals following i.p. injection of 150 mg/kg of D-luciferin potassium salt (Gold Biotechnology). Mice were anesthetized using isoflurane and images were acquired 10 minutes after injection. Signal intensity was quantified over a defined area of interest in the heads of the mice, as highlighted by the Living Image software.

### MRI analysis

To assess tumor volumes, MRI images were acquired using a 7-Tesla Bruker Biospec preclinical scanner. During scanning, the mice were kept sedated using an airflow of 1 to 1.5 mL/s with 1%–3% isoflurane. A gadolinium contrast agent was injected before all scans. The MRI scans included postcontrast T2-weighted (T2W) and postcontrast T1-weighted (T1W) imaging. The T2W scan was acquired with a rapid acquisition with relaxation enhancement (RARE) sequence, with a repetition time of 4,500 ms, echo time of 50 ms, RARE factor = 9, and number of averages = 4. The T1W scan was acquired with a RARE with short echo time (RAREst) sequence consisting of a repetition time of 819 ms, echo time of 4.6 ms, RARE factor = 4, and number of averages = 6. Both scans were collected with a field of view = 2 cm, slice thickness = 1 mm, number of slices = 12, and a matrix size of 128 × 128. Enhanced tumor volumes were manually segmented on the T1W and used for analysis.

### IHC

Coronal sections (30 μm) of brains were cut using a cryostat and sections were processed as required. Some sections were mounted on gelatinized Superfrost Plus slides for hematoxylin and eosin (H&E) staining; some were submerged in antifreeze solution (Nzytech) for free-floating immunofluorescence, and some were reserved for RNA extraction.

For H&E staining, sections were first placed in Harris’s hematoxylin solution (QCA), and sequentially placed in acid alcohol, distilled water wash, lithium carbonate solution, distillate water wash, and finally stained with 1% alcoholic eosin (QCA). Before mounting, sections were dehydrated in increasing concentrations of ethanol and finally rinsed in xylene before being mounted on slides.

For floating immunofluorescence staining, sections were washed with PBS to remove antifreeze solution and treated for antigen retrieval with 1% citrate solution at 80°C for 30 minutes. Sections were then blocked in PBS with 1% Triton X-100 (Sigma-Aldrich) and 5% BSA (Sigma-Aldrich) at room temperature prior to incubation with the relevant primary antibodies overnight at 4°C (Supplemental Table 3). Sections were then washed in PBS/0.1% Triton X-100, and incubated with Alexa Fluor–labeled secondary antibody (Invitrogen, Thermo Fisher Scientific). After an additional wash, they were mounted onto slides with 50% glycerol. Confocal fluorescence images were obtained using a Zeiss LSM 7 DUO confocal microscope.

For CD4, CD8, and FoxP3 staining, FFPE sections were deparaffinized and dehydrated through a standard ethanol series followed by blocking endogenous peroxidase activity using 1% hydrogen peroxide. Antigen retrieval using sodium citrate buffer pH 6.0 or Tris-EDTA pH 9.0 was performed followed by blocking sections with 10% normal goat serum before incubation with primary antibodies, followed by HRP-conjugated secondary antibody (ImmPRESS HRP horse anti–rabbit IgG Polymer Detection Kit, Vector Laboratories). The HRP-DAB polymer kit was used for visualization (ImmPACT DAB Substrate, Vector Laboratories). Slides were scanned using an Aperio AT2 slide scanner and analyzed using QuPath (v1.2.2) based on 5 randomly selected regions.

For GFAP staining, sections were incubated with polyclonal rabbit anti-GFAP antibody (1:500; Z0334, DAKO) overnight at 4°C. Sections were washed and incubated with goat anti-rabbit secondary antibody conjugated to Alexa Fluor 568 (1:200; Invitrogen) for 2 hours at room temperature. Sections were washed and incubated with prefiltered 0.1% Sudan black solution followed by additional washes. Slides were mounted using Vectashield antifade medium containing DAPI (Vector Laboratories) and were visualized using a Revolve microscope (ECHO).

### TME changes by immunostaining

For analysis of lipid-like body accumulation, we used Oil Red O staining. Formalin-fixed sections were stained with Oil Red O solution (Newcomer Supply) and hematoxylin according to the manufacturer’s protocol. After rinsing in distilled water, sections were counterstained with Gill’s III and mounted with Prolong Gold antifade reagent (Invitrogen). The formation of new blood vessels was assessed by the ratio of the proangiogenic marker α_v_β_3_ integrin to CD31 in the intratumoral section. For measurements of **˙**NO release in the TME, we double stained tumor-bearing brain sections with the microglial marker Iba-1 and 3-nitrotyrosine (3-NT), an indirect indicator of **˙**NO. The appearance of protein-linked 3-NT and colocalization with microglia/macrophages (Iba-1) was assessed using ImageJ software.

### qRT-PCR

RNA was extracted from FFPE brain sections using the FFPE-RNA Purification Kit (Norgen) and from cell pellets using the from RNeasy Kit (Qiagen). A Revert Aid First-Strand cDNA Synthesis Kit (Thermo Fisher Scientific) was used to convert 0.5 μg of total RNA into cDNA. The indicated primers (Supplemental Table 4) were used for qRT-PCR and performed using the LightCycler 480 (Roche Molecular Systems). Results were calculated using the delta Ct method and are represented as fold change compared to control.

### Microglial occupancy

Microglial occupancy was measure using ImageJ software. Microglia/macrophages were counted in a standard area of 10,000 μm^2^. Three sections per animal and 3 animals per group were analyzed. The counting was done exclusively inside the tumor, where GFP was clearly detected. Microglial occupancy was measured as percentage of pixels that surpass a constant threshold for Iba-1–Alexa Fluor 594 immunofluorescence. The data represent the percentage area inside the tumor that was immunoreactive for anti–Iba-1 antibody.

### In vitro studies

BV2 cells (1.5 × 10^5^) were plated in 6-well plates in triplicate wells and cultured for 24 hours before being treated with 100 ng/mL ADI-PEG20. Six hours after ADI-PEG20 treatment, cells were irradiated at various doses and harvested 18 hours later for RNA extraction.

For studies looking at the effects of GL261-conditioned media (CM) on BV2 cells, CM was collected from an overnight culture of GL261 cells that were at no more than 70% confluence. Cellular debris from the CM was removed by centrifugation at 9000*g* before passing through a 0.22 μm filter and stored at −80°C until required. The CM was prewarmed and added to exponentially growing BV2 microglial cells with or without 1 mg/mL ADI-PEG20 for 6 hours. RNA was extracted from BV2 cell pellets and assessed by qPCR for *Arg1* expression levels.

### Bioinformatic analysis

A list of genes involved in microglial polarization was curated from published literature (ref. 33 and Supplemental Table 1). The resulting list of genes was matched to TCGA Affymetrix microarray data, which resulted in a list of 17 genes available for analysis. Microarray data were quantile normalized and *z*-scores were generated for each gene. Hierarchical clustering (Euclidean distance, Ward method) was performed on the gene set. Phenotypic data such as sample source and GBM subtype were included to identify any correlations.

### Statistics

Unless otherwise specified, data were analyzed using GraphPad Prism (v9.3.1). For 2 groups, Student’s *t* test was used (2-tailed). For more than 2 groups, 1-way ANOVA with Tukey’s multiple comparison test was used, and adjusted *P* values are reported. For all box-and-whisker plots, horizontal lines within boxes denote median values, boxes extend from the 25th to the 75th percentile of each group's distribution of values, and bottom whiskers represent the 10th percentile and the top whiskers represent the 90% percentile. Dots denote values outside this range. Some plots have no data points outside this range because of limited sample size. In these plots, the whiskers represent the lowest and highest value in the data set. Statistical significance was defined as *P* less than 0.05.

### Study approval

The study was approved by Imperial College London Research and Ethics Committee (REC 14/EE/0024). All experiments conducted at the University of Seville were approved by the University of Seville Ethical Committee for Experimental Research and fulfilled the requirements for experimental animal research in accordance with the Guidelines of the European Union Council (86/609/EU) and the Spanish regulations (BOE 34/11370–421, 2013) for the use of laboratory animals.

The St. Joseph Hospital and Medical Center’s Institutional Animal Care and Use Committee approved of all experimental procedures performed at the Barrow Neurological Institute and all animals were treated humanely in accordance with the Laboratory Animal Welfare Act (Animal Welfare Assurance no. A3519-01, protocol 575).

## Author contributions

NH performed the in vitro experiments and the GFP in vivo experiments, assisted in drafting the first version of the manuscript, and generated first versions of most of the figures. JGR and MSS performed the in vivo survival studies and carried out IHC analysis and generated some figures. NH, JGR, and MSS share co–first authorship and justification of assigning authorship order is as follows: NH started the study by performing the vitro and GFP in vivo studies, and JGR did the in vivo experiments using bioluminescence under the direction of MSS and NS. JGR and MSS did the in vivo analysis and some of the IHC. RP performed the bioinformatic analysis, and helped to edit the manuscript and some figures. JS performed the IHC analysis for CD4, CD8, and FoxP3, H&E analysis on the CT-2A mouse tissues, and generated the figures for these mice. MLOV and SMC assisted with the in vivo radiation experiments using the GL261 tumor model and helped to edit the manuscript. DRH performed the in vivo experiments using the CT-2A tumor model. CCQ and YG performed the MRI analysis. ACS provided the GL261-Luc2 cells, assisted with the design of the CT-2A in vivo experiments, and made final edits to the figures and together with KS, JB, AGT, TC, GA, and KON provided valuable editorial contributions to the manuscript. KS provided the CT-2A cells. AGT provided insightful chemical rationale and analysis as well as discussions on ways to manipulate the TME. JLV provided invaluable discussions on the involvement of the TME, and made major contributions to the editing and construction of the figures. NS initiated and supervised the overall study, was responsible for the experimental designs, editing, and writing the final version of the manuscript.

## Supplementary Material

Supplemental data

## Version 1 02/03/2022

In-Press Preview

## Version 2 03/15/2022

Electronic publication

## References

[1] Stupp R (2005). Radiotherapy plus concomitant and adjuvant temozolomide for glioblastoma. N Engl J Med.

[2] Stupp R (2009). Effects of radiotherapy with concomitant and adjuvant temozolomide versus radiotherapy alone on survival in glioblastoma in a randomised phase III study: 5-year analysis of the EORTC-NCIC trial. Lancet Oncol.

[3] Weller M (2019). How we treat glioblastoma. ESMO Open.

[4] Lukas RV (2019). Newly diagnosed glioblastoma: a review on clinical management. Oncology (Williston Park).

[5] Schiffer D (2017). A comprehensive view of tumor stem cells and their regulation by the microenvironment in glioblastoma. Neurol Sci.

[6] Russo CD, Cappoli N (2018). Glioma associated microglia/macrophages, a potential pharmacological target to promote antitumor inflammatory immune response in the treatment of glioblastoma. Neuroimmunol Neuroinflammation.

[7] Roesch S (2018). When immune cells turn bad-tumor-associated microglia/macrophages in glioma. Int J Mol Sci.

[8] Andreou KE (2017). Anti-inflammatory microglia/macrophages as a potential therapeutic target in brain metastasis. Front Oncol.

[9] Lisi L (2017). Expression of iNOS, CD163 and ARG-1 taken as M1 and M2 markers of microglial polarization in human glioblastoma and the surrounding normal parenchyma. Neurosci Lett.

[10] Husson A (2003). Argininosuccinate synthetase from the urea cycle to the citrulline-NO cycle. Eur J Biochem.

[11] Haines RJ (2011). Argininosuccinate synthase: at the center of arginine metabolism. Int J Biochem Mol Biol.

[12] Shresththa S (2018). Nitric oxide: it’s role in immunity. J Clin Diagn Res.

[13] Casero RA (2018). Polyamine metabolism and cancer: treatments, challenges and opportunities. Nat Rev Cancer.

[14] Phang JM (2019). Proline metabolism in cell regulation and cancer biology: Recent advances and hypotheses. Antioxid Redox Signal.

[15] Wu SY, Watanabe K (2017). The roles of microglia/macrophages in tumor progression of brain cancer and metastatic disease. Front Biosci (Landmark Ed).

[16] Fumagalli M (2018). How to reprogram microglia towards beneficial functions. Glia.

[17] Rabinovitch S (2015). Diversion of aspartate in ASS1-deficient tumours fosters de novo pyrimidine synthesis. Nature.

[18] Keshet R (2018). Rewiring urea cycle metabolism in cancer to support anabolism. Nat Rev Cancer.

[19] Syed N (2013). Epigenetic status of argininosuccinate synthetase and argininosuccinate lyase modulates autophagy and cell death in glioblastoma. Cell Death Dis.

[20] Przystal JM (2018). Efficacy of arginine depletion by ADI-PEG20 in an intracranial model of GBM. Cell Death Dis.

[21] Maggi M, Scotti C (2019). Enzymes in metabolic anticancer therapy. Adv Exp Med Biol.

[22] Hall PE (2019). A phase I study of pegylated arginine deiminase (pegargiminase), cisplatin, and pemetrexed in argininosuccinate synthetase 1-deficient recurrent high-grade glioma. Clin Cancer Res.

[23] Lind DS (2004). Arginine and Cancer: arginine metabolism: enzymology, nutrition and clinical significance. J Nutr.

[24] Morris SM (2006). Arginine: beyond protein. Am J Clin Nutr.

[25] Geiger R (2016). L-Arginine modulates T cell metabolism and enhances survival and anti-tumour activity. Cell.

[26] Werner A (2017). Reconstitution of T cell proliferation under arginine limitation: activated human T cells take up citrulline via L-type amino acid transporter 1 and use it to regenerate arginine after induction of argininosuccinate synthase expression. Front Immunol.

[27] Szabó C (2007). Peroxynitrite: biochemistry, pathophysiology and development of therapeutics. Nat Rev Drug Discov.

[28] Abdelwahab MG (2011). Intracranial implantation with subsequent 3D in vivo bioluminescent imaging of murine gliomas. J Vis Exp.

[29] Fraszczak J (2010). Peroxynitrite-dependent killing of cancer cells and presentation of released tumor antigens by activated dendritic cells. J Immunol.

[30] Kumar A (2014). Inducible nitric oxide synthase is key to peroxynitrite-mediated, LPS-induced protein radical formation in murine microglial BV2 cells. Free Radic Biol Med.

[31] Heijnen HF (2006). Subcellular localization of tyrosine-nitrated proteins is dictated by reactive oxygen species generating enzymes and by proximity to nitric oxide synthase. Free Radic Bio Med.

[32] Orihuela R (2016). Microglial M1/M2 polarization and metabolic states. Br J Pharmacol.

[33] Jun-ichi S (2018). Gene expression profiles of M1 and M2 microglia characterized by comparative analysis of public datasets. Clin Exp Neuroimmunol.

[34] Martínez-Murillo R, Martínez A (2007). Standardization of an orthotopic mouse brain tumor model following transplantation of CT-2A astrocytoma cells. Histol Histopathol.

[35] Park H (2008). Arginine deiminase enhances MCF-7 cell radiosensitivity by inducing changes in the expression of cell cycle-related proteins. Mol Cells.

[36] Burrows N (2016). Hypoxia-induced nitric oxide production and tumour perfusion is inhibited by pegylated arginine deiminase (ADI-PEG20). Sci Rep.

[37] Wang G (2017). Advances in the targeting of HIF-1α and future therapeutic strategies for glioblastoma multiforme. Oncol Rep.

[38] Lo Dico A (2018). Hypoxia-inducible factor-1α activity as a switch for glioblastoma responsiveness to temozolomide. Front Oncol.

[39] Mori M (2007). Regulation of nitric oxide synthesis and apoptosis by arginase and arginine recycling. J Nutr.

[40] Baydoun AR (1994). Discrimination between citrulline and arginine transport in activated murine macrophages: inefficient synthesis of NO from recycling of citrulline to arginine. Br J Pharmacol.

[41] Radi R (2018). Oxygen radicals, nitric oxide, and peroxynitrite: Redox pathways in molecular medicine. Proc Natl Acad Sci U S A.

[42] Ferrer-Sueta G, Radi R (2009). Chemical biology of peroxynitrite: kinetics, diffusion, and radicals. ACS Chem Biol.

[43] Jay-Gerin JP, Ferradini C (2000). Are there protective enzymatic pathways to regulate high local nitric oxide (NO) concentrations in cells under stress conditions?. Biochimie.

[44] Azzam EI (2012). Ionizing radiation-induced metabolic oxidative stress and prolonged cell injury. Cancer Lett.

[45] Ensor CM (2002). Pegylated arginine deiminase (ADI-SS PEG20,000 mw) inhibits human melanomas and hepatocellular carcinomas in vitro and in vivo. Cancer Res.

[46] Roe ND, Ren J (2012). Nitric oxide synthase uncoupling: a therapeutic target in cardiovascular diseases. Vascul Pharmacol.

[47] Zhang I (2016). Characterization of arginase expression in glioma-associated microglia and macrophages. PLoS One.

[48] Kmiecik J (2013). Elevated CD3^+^ and CD8^+^ tumor-infiltrating immune cells correlate with prolonged survival in glioblastoma patients despite integrated immunosuppressive mechanisms in the tumor microenvironment and at the systemic level. J Neuroimmunol.

[49] Behnan J (2019). The landscape of the mesenchymal signature in brain tumours. Brain.

[50] Hajji N (2010). Opposing effects of hMOF and SIRT1 on H4K16 acetylation and the sensitivity to the topoisomerase II inhibitor etoposide. Oncogene.

