# Supplementary material for: Arginine deprivation alters microglial polarity and synergizes with radiation to eradicate non-arginine-auxotrophic glioblastoma tumors
Source: J Clin Invest. 2022 Mar 15;132(6):e142137. doi: 10.1172/JCI142137 (PMC8920336; doi:10.1172/JCI142137)
Supplement: Supplemental data [file jci-132-142137-s216.pdf]

Supplementary Figures and Legends

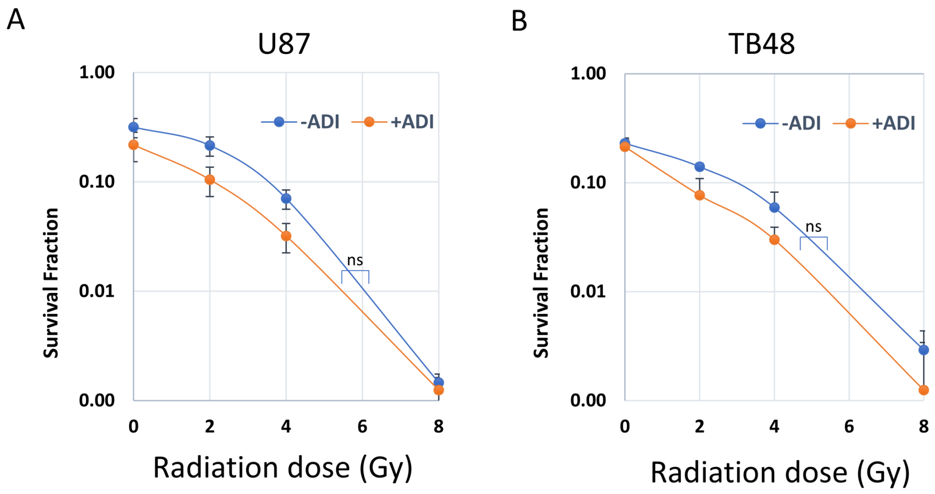

Supplementary Figure 1 (S1): ADI-PEG20 does not potentiate the effects of radiation in 2D cultures: Replicate plates of established and primary ASS1 positive GBM cells U87 and TB48, respectively were pre-incubated with ADI-PEG20 (1µg/ml) for 24h then treated with different doses of ionising radiation. After 14 days of growth, plates were fixed, stained with crystal violet and the number of surviving colonies were plotted as means ± SD, n=12 (A-B). Statistical analysis was performed using two-way ANOVA with Bonferroni post-test using GraphPad Prism, ns- non-significant

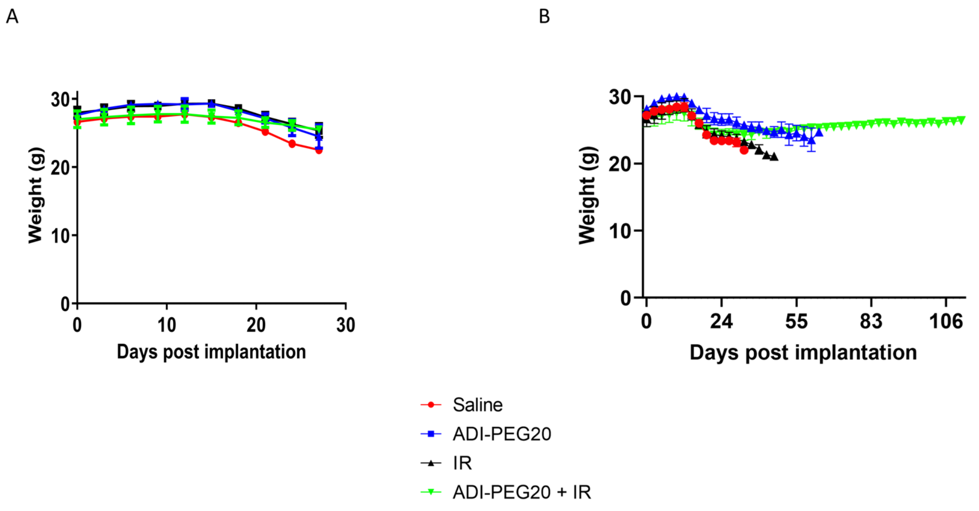

Supplementary Figure 2 (S2): Average weight of mice with GL261 tumors (g) during the course of the study.

A: Mice harbouring GL261-luc2 tumors harvested at day 28

B: Mice harbouring GL261-luc2 tumors, survival study

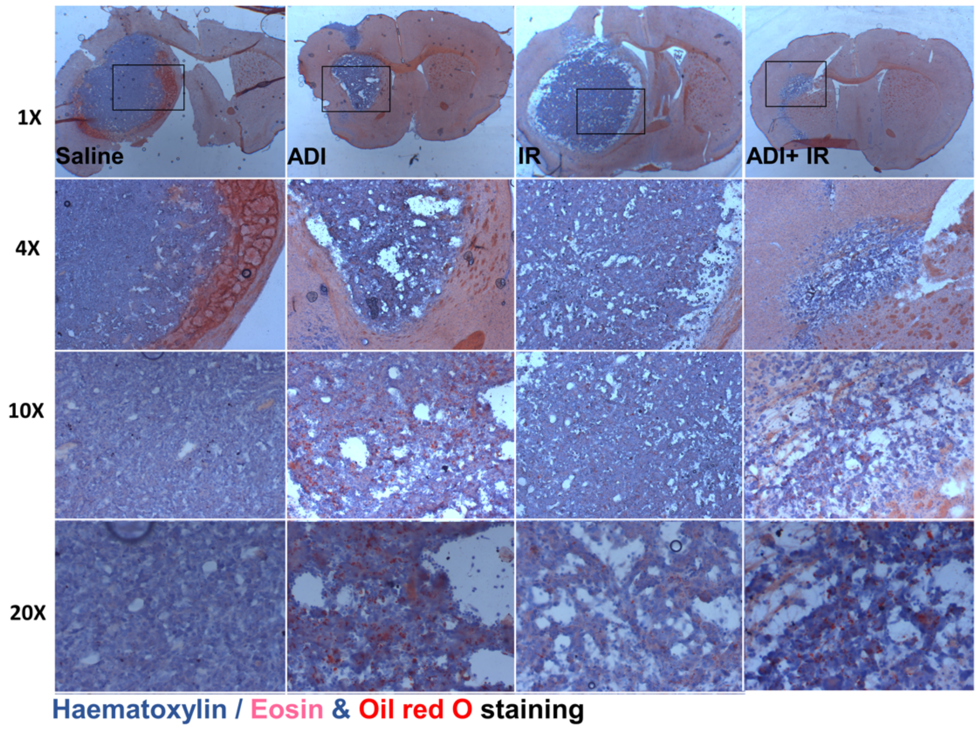

Supplementary Figure 3 (S3): Phagocytic activity of microglia in response to arginine deprivation

Immunohistochemical assessment of microglial phagocytic capacity by Oil red O staining of representative mice sections at different magnifications.

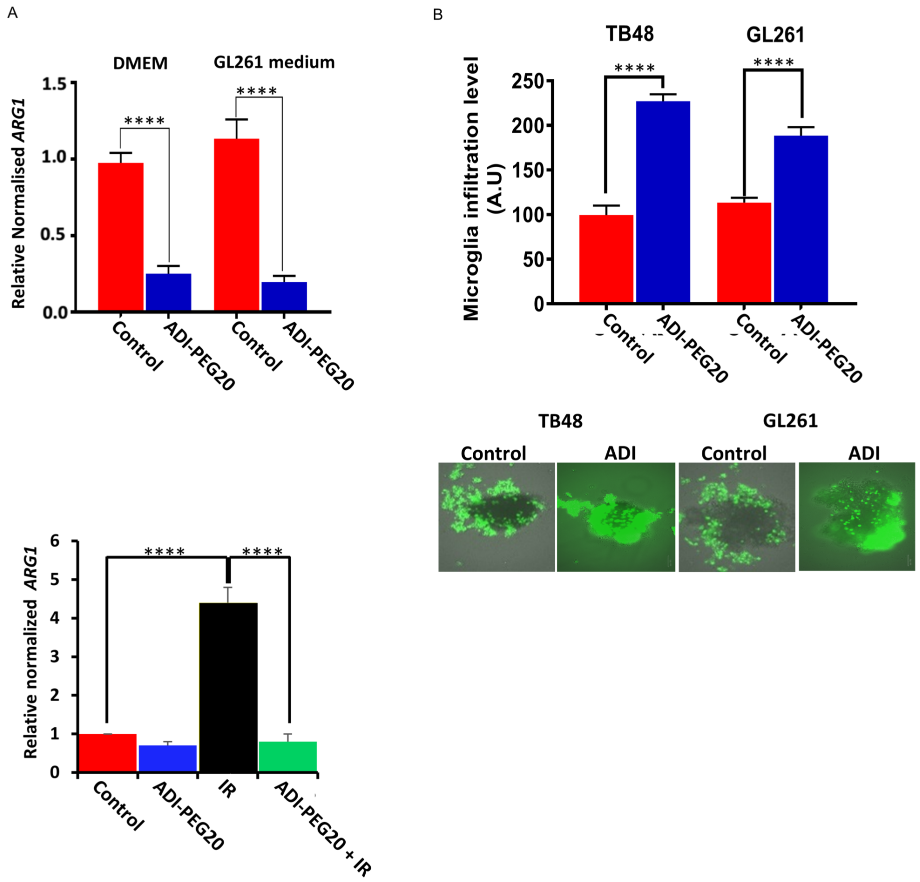

Supplementary Figure 4 (S4): In vitro analysis of Arg1 expression in microglia cells

In vitro assessment of microglial (BV2 cells) expression of Arg1 when cultured in fresh media or conditioned media from tumour cells (GL261) treated with ADI-PEG20 (1µg/ml) for 24 hours (A). Infiltration of microglia in co-cultures of GBM neurospheres (GL261 and TB48) and microglia (GFP expressing BV2 cells) treated with ADI-PEG20 for 24hours (B). In vitro assessment of microglial (BV2 cells) expression of *Arg1* after treatment with ADI-PEG20 (100ng/ml), radiation and ADI-PEG20 + radiation (C). Results are the means +/- SEM of two experiments (n=3) analysed in triplicate using One-way ANOVA. * *p* <0.05, ** *p* <0.01, *** *p* <0.001, **** *p* <0.0001.

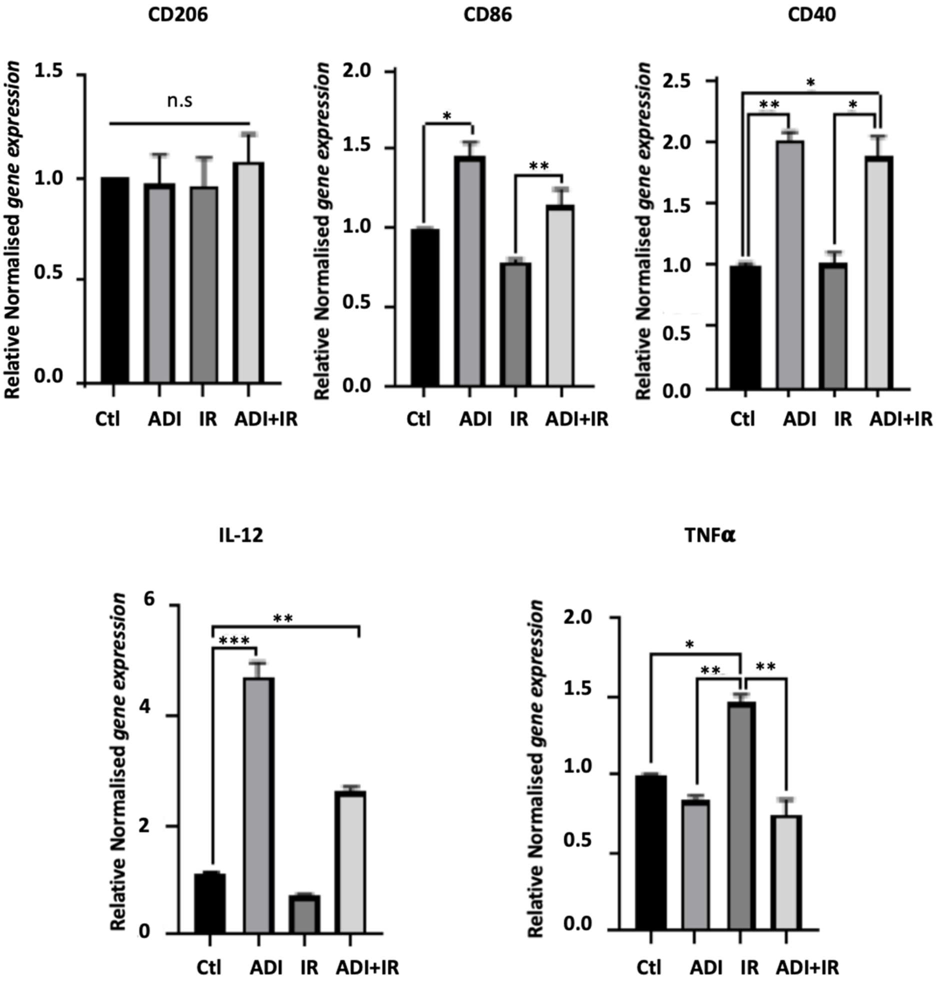

Supplementary Figure 5 (S5): Microglia expression of pro-inflammatory markers in response to arginine deprivation in vitro.

Relative gene expression and secretion of pro-inflammatory markers and cytokines in BV2 cells in response to ADI-PEG-20 (100ng/ml), radiation (2Gy) monotherapy and in combination (B). Data represents the mean +/- SEM (n=2) analysed using One-way ANOVA. * p <0.05, ** p <0.01, *** p <0.001, **** p <0.0001

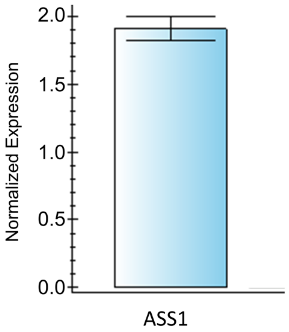

Supplementary Figure 6 (S6): qPCR analysis of ASS1 mRNA expression in CT-2A cells

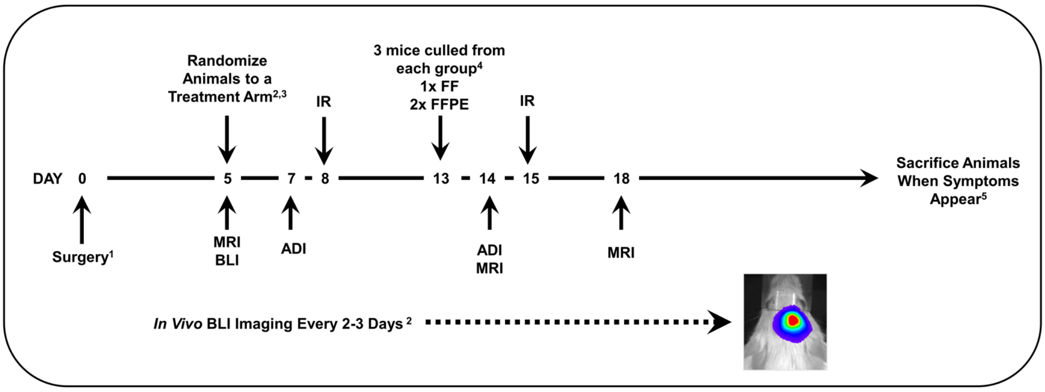

1 Intracranial implantation of CT-2A-luc cells into 10 week-old C57BL/6 female albino mice (average weight 20g)

[2] Animals are given a subcutaneous injection of luciferin and imaged under isofluorane anesthesia on a warmed platform using the IVIS® Spectrum *in vivo* imaging system.

[3] Animals are randomized to a treatment arm – 10 animals per treatment arm, per experiment: ADI-PEG20 treated animals received 5IU administered via intramuscular injection every 7 days starting from day 7 after implantation. Radiation (4Gy of whole brain radiation) given 16hrs after ADI-PEG20 treatment on a weekly basis.

[4] FF = flash frozen; FFPE = formalin-fixed paraffin embedded

[5] Animals are sacrificed when a reproducible set of symptoms appear which include lethargy, failure to ambulate, anorexia resulting in >10% loss of weight.

Supplementary Figure 7: Schematic diagram depicting the experimental protocol for in vivo experiments using CT-2A cells/tumors

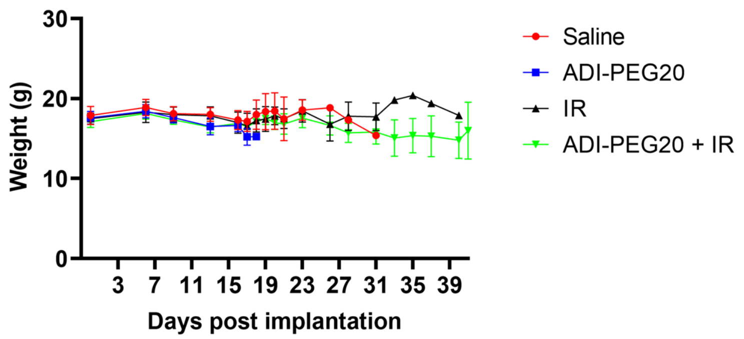

Supplementary Figure 8: Average weight of mice harbouring CT-2A tumors during the course of the study. Vertical bars are SD.

Gene	Description	In TCGA	Reference
ARG1	Arginase 1	Yes	doi: 10.3390/ijms19020436 and ” Nature Reviews Immunology, vol. 5, no. 8, pp. 641–654, 2005.
LLGL1 (MGL1)	LLGL Scribble Cell Polarity Complex Component 1	Yes	doi: 10.1016/j.smim.2015.10.003.
ITGB3	Integrin subunit beta 3	Yes	Cell. 1999 Feb 5;96(3):319-28. and Perspect Medicin Chem. 2008; 2: 57–73.
TGFB1	Transforming growth factor beta 1	Yes	doi: 10.1007/s10555-010-9226-3 and dOI:https://doi.org/10.1016/j.cell.2008.07.001
CTLA4	Checkpoints T-lymphocyte-associated protein 4	Yes	doi: 10.1007/s11060-015-1747-8 and doi: 10.3389/fonc.2018.00086
CCR2	CC chemokine receptor 2	Yes	https://doi.org/10.1073/pnas.1604263113
COL14A1	Collagen type XIV alpha 1 chain	Yes	doi: 10.1158/1541-7786.MCR-13-0236
TREM2	Triggering receptor expressed on myeloid cells 2	Yes	doi: 10.1586/1744666X.2015.1043893
FPR1	Formylpeptide receptor1 (FPR1)	Yes	doi: 10.1016/j.intimp.2012.07.015
CLEC7A	C-Type Lectin Domain Containing 7A	Yes	doi: 10.3389/fimmu.2018.00227 and doi: 10.1186/s12974-020-01797-2.
MS4A6A (MS4A6B)	Membrane spanning 4-domains A6A	Yes	Neurobiol Aging. 2014 Feb;35(2):279-90. doi: 10.1016/j.neurobiolaging.2013.08.002. Epub 2013 Sep 21. and doi: 10.1016/j.redox.2017.10.01
CD163	CD163 Molecule	Yes	doi: 10.1089/ars.2012.4834 and https://doi.org/10.1080/2162402X.2019.1601478
AQP9	Aquaporin 9	Yes	doi: 10.3390/ijms17071029 and J Transl Med. 2019 Nov 8;17(1):363. doi: 10.1186/s12967-019-2113-y.
ITGAM	Integrin subunit alpha M	Yes	doi: 10.1038/nrc2748 and Cell. 1999 Feb 5;96(3):319-28. and doi: 10.3892/ol.2016.4626
IL33	Interleukin 33	Yes	doi: 10.1007/s12079-018-0464-4
CHI3L1	Chitinase 3 like 1	Yes	doi: 10.1007/s12026-013-8459-y and Oncogene volume 31, pages3111–3123(2012)
CHI3L2	Chitinase 3 like 2	Yes	doi: 10.1007/s12026-013-8459-y and doi: 10.21873/anticanres.13915 Anticancer Research

Supplementary Table 1: Microglia/macrophage pro-tumour gene list generated from published manuscripts

	Cluster	
Verhaak subtype	1	2
Classical	-2.538 (8.96)	3.091 (13.30)
Mesenchymal	3.255 (14.74)	-3.964 (21.87)
Neural	2.224 (6.88)	-2.708 (10.21)
Proneural	-2.637 (9.68)	3.212 (14.36)

Supplementary Table 2: **GBM subtypes are specifically associated with the pro-tumour immune signature.** Table showing the results of a Chi-Squared test between GBM subtypes and immune class. Residuals with contributions shown in brackets (%)

PRIMARY ANTIBODY	COMPANY
Mouse anti-$a_v\beta_3$ (ab238667)	Abcam (Cambridge, UK)
Goat anti-CD31/PECAM-1 (AF3387)	R&D Systems, Bio-Techne (Minneapolis, MN, USA)
Rabbit anti-GFAP (RP014)	Diagnostic BioSystems (Pleasanton, CA, USA)
Mouse anti-Iba1 (MABN92)	Millipore, Merck (Darmstadt, Germany)
Mouse anti-3-NT (ab110282)	Abcam (Cambridge, UK)
Rabbit anti-γH2AX (9718)	Cell Signaling Technology (Danvers, MA, USA)
Goat anti-Arg1 (IMG-30305)	NOVUS Biologicals, Bio-techne (Minneapolis, MN, USA)
Rabbit anti-iNOS (ab178945)	Abcam (Cambridge, UK)
Rabbit anti-CD4 (93518)	Cell Signaling Technology (Danvers, MA, USA)
Rabbit anti-CD8 (ab217344)	Abcam (Cambridge, UK)
Rabbit anti-FoxP3 (ab215206)	Abcam (Cambridge, UK)
Rabbit anti-GFAP (Z033429-2)	Agilent Technologies LDA UK Limited

Supplementary Table 3: Primary antibodies

Human primers

Gene	Forward	Reverse
GFP	CACTACTTGTCCACCCAATCTG	GCAGCGGTCACAAACTCAAG
Arg1	GTTGATGTCCCTAATGACAGC	CATTCTTCTGGACCTCTGCC
NOS2	GAGCCACAGTCCTCTTTGC	CTCTCTTGCGGACCATCTCC
Ym1	GTACCCTGGGTCTCGAGGAA	GCCTTGGAATGTCTTTCTCCAC
TNFα	TGCCTATGTCTCAGCCTCTTC	GAGGCCATTTGGGAACTTCT
β-actin	GGCTATGCTCTCCCTCACG	CTTCTCTTTGATGTCACGCACG

Supplementary Table 4: Human qPCR primer sequences
